# Structural insights into curdlan degradation via a glycoside hydrolase containing a disruptive carbohydrate-binding module

**DOI:** 10.1186/s13068-024-02494-5

**Published:** 2024-03-21

**Authors:** Tianhang Lv, Juanjuan Feng, Xiaoyu Jia, Cheng Wang, Fudong Li, Hui Peng, Yazhong Xiao, Lin Liu, Chao He

**Affiliations:** 1 School of Life Sciences and Anhui Key Laboratory of Modern Biomanufacturing, Anhui University, Hefei, Anhui China; 2 MOE Key Laboratory for Cellular Dynamics, School of Life Sciences, Division of Life Sciences and Medicine, University of Science and Technology of China, Hefei, China

**Keywords:** β-1,3-Glucanase, Carbohydrate-binding module, Curdlan, Triple-helical β-1,3-glucan

## Abstract

**Background:**

Degradation via enzymatic processes for the production of valuable β-1,3-glucooligosaccharides (GOS) from curdlan has attracted considerable interest. CBM6E functions as a curdlan-specific β-1,3-endoglucanase, composed of a glycoside hydrolase family 128 (GH128) module and a carbohydrate-binding module (CBM) derived from family CBM6.

**Results:**

Crystallographic analyses were conducted to comprehend the substrate specificity mechanism of CBM6E. This unveiled structures of both apo CBM6E and its GOS-complexed form. The GH128 and CBM6 modules constitute a cohesive unit, binding nine glucoside moieties within the catalytic groove in a singular helical conformation. By extending the substrate-binding groove, we engineered CBM6E variants with heightened hydrolytic activities, generating diverse GOS profiles from curdlan. Molecular docking, followed by mutation validation, unveiled the cooperative recognition of triple-helical β-1,3-glucan by the GH128 and CBM6 modules, along with the identification of a novel sugar-binding residue situated within the CBM6 module. Interestingly, supplementing the CBM6 module into curdlan gel disrupted the gel’s network structure, enhancing the hydrolysis of curdlan by specific β-1,3-glucanases.

**Conclusions:**

This study offers new insights into the recognition mechanism of glycoside hydrolases toward triple-helical β-1,3-glucans, presenting an effective method to enhance endoglucanase activity and manipulate its product profile. Furthermore, it discovered a CBM module capable of disrupting the quaternary structures of curdlan, thereby boosting the hydrolytic activity of curdlan gel when co-incubated with β-1,3-glucanases. These findings hold relevance for developing future enzyme and CBM cocktails useful in GOS production from curdlan degradation.

**Supplementary Information:**

The online version contains supplementary material available at 10.1186/s13068-024-02494-5.

## Background

Curdlan is an exopolysaccharide that is insoluble in water. It consists of glucose residues linked in a linear β-1,3 configuration and is naturally synthesized by specific bacterial and fungal species. Curdlan forms single and triple-helical structures. By heating at above 80 ℃, curdlan generates thermal irreversible gels (high-set gels) that exhibit a predominantly triple-helical structure [1]. Curdlan is widely applied in the food and pharmaceutical industries [2]. However, curdlan’s insolubility in water limits its direct applications. Its hydrolysate, known as β-1,3-glucooligosaccharides (GOS), exhibits enhanced water solubility and demonstrates improved biological activities such as immunostimulatory [3, 4], antimicrobial [5, 6], and prebiotic properties [7, 8]. Moreover, the degree of polymerization (DP) of GOS influences its bioactivity [9]. Therefore, curdlan serves as an economical source for the production of GOS.

The enzymatic degradation of curdlan has gained considerable attention due to its environmentally friendly characteristic and high-yield potential. The curdlan-hydrolyzing enzymes previously documented are distributed across various glycoside hydrolase families, including GH16, GH50, GH64, GH81, GH128, GH157, and GH158 [10–15]. Enhanced comprehension of the mechanism governing enzymatic recognition and cleavage of triple-helical β-1,3-glucan would significantly propel the molecular engineering of enzymes capable of curdlan degradation. However, the mechanism of how a glycoside hydrolase recognizes and hydrolyzes β-1,3-glucan with a triple-helical quaternary structure remains controversial until now. Two examples involve *Paenibacillus barengoltzii* GH64 β-1,3-glucanase (PbBgl64A) and *Bacillus halodurans* GH81 β-1,3-glucanase (BhGH81), where the crystal structures revealed the presence of two or three GOS chains in their active sites, overlapping with two separate chains in the triple-helical β-1,3-glucan molecule [16, 17]. Therefore, these two examples implied that GHs could recognize the entire triple helices in the catalytic groove. Recently, another example proposes a curdlan-unwinding model during hydrolysis, utilizing molecular docking studies and examining the conformation of differently treated curdlan along with the hydrolysis rate by various β-1,3-glucanases, including PbBgl64A [18]. According to this proposal, recognition of triple-helical curdlan occurs through interactions with certain ancillary regions of β-1,3-glucanases, preceding the unwinding of curdlan into single- and double-helical forms. These forms then fit more appropriately into the catalytic cavity, where the polysaccharide chains are eventually hydrolyzed into GOS. Therefore, more structural accounts of β-1,3-glucanase-catalyzed curdlan hydrolysis are needed to clarify the mechanism.

Our previous work revealed a curdlan-specific endo-β-1,3-glucanase, CBM6E, from a marine bacterium *Saccharophagus degradans* 2-40^T^, which contains an N-terminal GH128 module and a C-terminal CBM6 module. CBM6E exhibits catalytic activity at intermediate temperatures and efficiently produces GOS with DPs of 3–6 from curdlan. The CBM6 module of CBM6E (CBM6E-CBM6) exhibits a strong binding affinity specifically to curdlan, effectively enhancing the enzyme’s activity against curdlan [19]. These characteristics render CBM6E highly appealing for the production of GOS. However, its curdlan recognition mechanism has yet to be elucidated. Furthermore, many GH128 modules are fused with CBMs from the CBM4 or CBM6 family [20]. However, the structures of tandem GH128 and CBM modules remain unknown.

In this study, we solved the crystal structures of CBM6E in both the apo form (2.4 Å) and complexed with GOS (1.28 Å). This represents the first structure of a tandem GH128 and CBM6 module. Based on the complex structure, we designed mutants of CBM6E with enhanced hydrolytic activities and different product spectra toward curdlan. Furthermore, we conducted molecular docking, site-directed mutagenesis, enzymatic kinetics, and curdlan binding assays to identify ancillary binding sites for triple-helical β-1,3-glucan, particularly within the CBM6 module. Additionally, we unveiled a novel function of the CBM6 module in disrupting the microstructure of curdlan, as demonstrated through Congo red staining and scanning electron microscopy (SEM). We further conducted assays to explore whether the production of reducing ends, resulting from the hydrolysis of curdlan by specific β-1,3-glucanase, is amplified upon the pre-addition of the disruptive CBM6 module to the curdlan substrate.

## Results

### GH128 and CBM6 fold as a whole

**Fig. 1:**
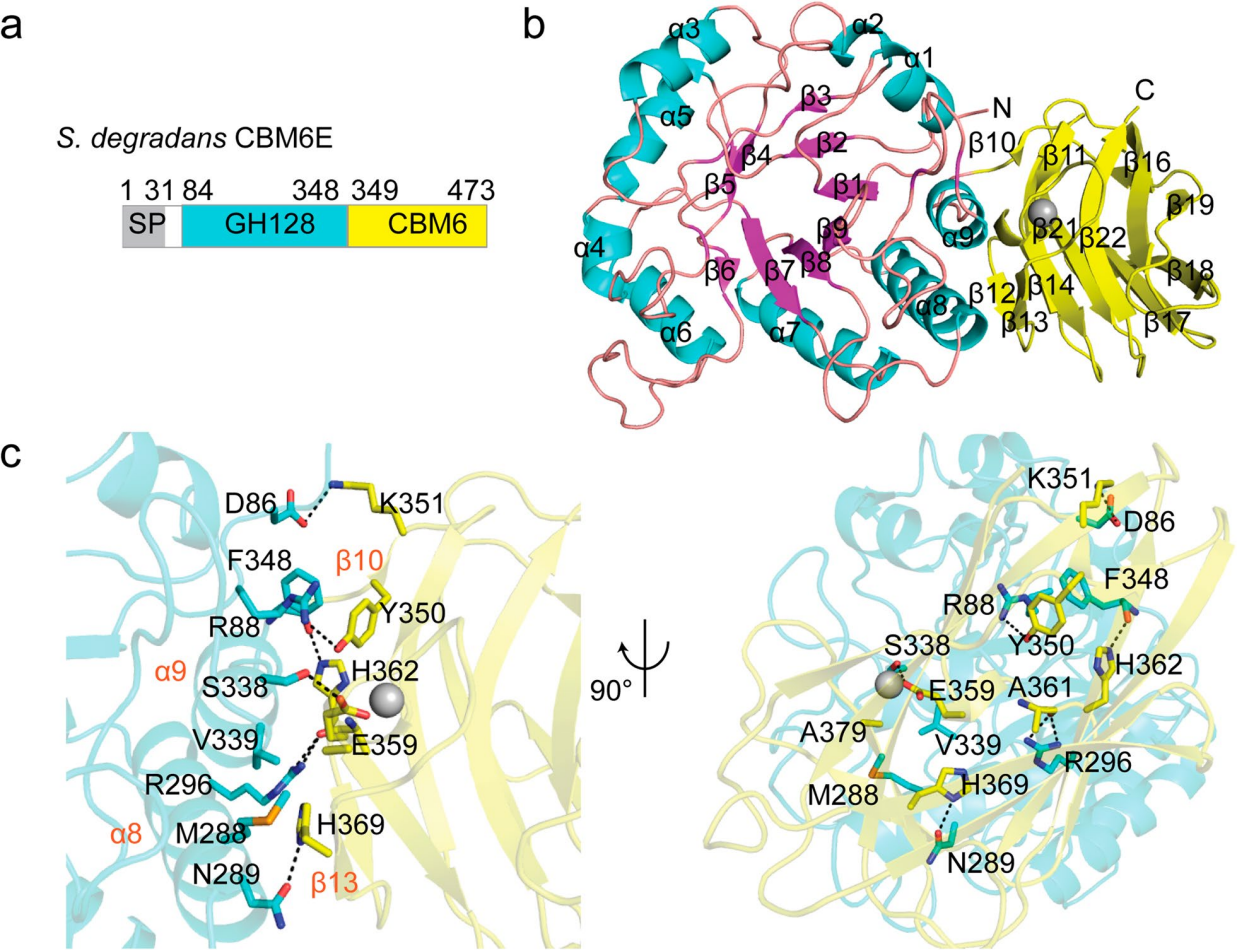
Overall structure of CBM6E. **a** Domain architecture of CBM6E. The CBM6E protein contains a tandem arrangement of two domains: GH128 and CBM6. SP, signal peptide. **b** Overall structure of the tandem GH128 and CBM6 domain of CBM6E. The catalytic GH128 module adopts an (α/β) barrel scaffold, where the α-helices are depicted in magenta and the β-sheet in cyan. On the other hand, the CBM6 module adopts a common β-sandwich fold, which is colored yellow. **c** Interdomain interface between GH128 (cyan) and CBM6 (yellow). Residues that participate in forming contacts between the two domains are represented using a stick representation. Hydrogen bonds, indicated by dashed lines, are formed between specific residues with a distance of less than 3.5 Å

The crystal structure of CBM6E was determined at a resolution of 2.4 Å. The structure of CBM6E comprises two distinct domains: an (α/β)-barrel catalytic domain (residues 84–348) belonging to the GH128 family, and a C-terminal β-sandwich domain (residues 349–473) belonging to the CBM6 family (Fig. 1a). The CBM6 domain forms an interdomain interface with GH128 primarily through hydrogen bonds between α8 and α9 of the GH128 domain and β10 and β13 of the CBM6 domain (Fig. 1b). Notably, the interdomain interface involves the following residues: D86, K351, R88, Y350, N289, H369, R296, A361, S338, E359, F348, and H362, with each pair forming hydrogen bonds. Additionally, R88, F348, Y350, M288, V339, and A379 participate in hydrophobic interactions as trios (Fig. 1c). Using the ConSurf web server, we identified that most of the residues at the interdomain interface are evolutionarily conserved (Additional file 1: Fig. S1). This conservation suggests a certain degree of evolutionary stability in the folding of GH128 and CBM6 as a cohesive unit.

### Structural comparison

We conducted a comprehensive structural comparison of CBM6E with entries in the Protein Data Bank (PDB) using the DALI server. The analysis revealed that the closest structural neighbors belonged to subgroup II of the GH128 family: ScGH128_II (PDB code 6uax) and PvGH128_II (PDB code 6uav) [20]. ScGH128_II and PvGH128_II exhibited sequence identities of 42% and 39%, respectively, with the GH128 domain of CBM6E. Regarding structural similarity, ScGH128_II displayed an RMSD value of 1.5 Å over 241 matched Ca atoms, while PvGH128_II had an RMSD value of 1.7 Å over 247 matched Ca atoms. In addition, this analysis indicated that the CBM6 domain of CBM6E shared similarities with ZgLamC_CBM6_ (PDB code 5fui), a marine CBM6 module known for its ability to bind laminarin [21]. The structural alignment revealed an RMSD value of 1.5 Å over 117 matched Ca atoms, accompanied by a sequence identity of 22%. Notably, we observed a Mg^2+^ ion within CBM6E-CBM6, occupying a similar position as seen in ZgLamC_CBM6_ (Additional file 1: Fig. S2a). This ion demonstrated an six-coordinate octahedral geometry and coordinated with Glu357 OE1, Glu359 OE1, Ala379 O, Asn467 OD, Asn467 O, and a water molecule (Additional file 1: Fig. S2b). The Mg^2+^ complex was supported by its six-coordinate octahedral geometry and B factors consistent with neighboring atoms. Furthermore, upon modeling a Ca^2+^ ion, which similarly adopted a six-coordinate octahedral geometry, into the same site, it led to a negative (red) Fo–Fc map for the ion after crystal structure refinement (Additional file 1: Fig. S2c).

This DALI search also yielded some tandem GH and CBM modules, such as *Geobacillus stearothermophilus* β-d-xylosidase XynB1 (PDB code 2bfg) [22] and *Pseudomonas aeruginosa* PslG (PDB code 4zn2) [23]. However, the relative orientations between the GH and CBM domains in these proteins totally differ from what was observed in CBM6E. The distinct directional arrangement of the GH and CBM domains may not only influence the stability of the protein comprising these domains but also play a crucial role in the coordinated recognition of specific polysaccharide substrates by these domains, a phenomenon suggested for certain multimodular glycoside hydrolases [24]. Additionally, there are instances where the binding of a CBM to a carbohydrate ligand and the accommodation of a substrate in the active site of the catalytic module do not exhibit coordinated movements, often characterized by flexible interdomain orientations [24, 25].

### Negative-subsite region

**Fig. 2:**
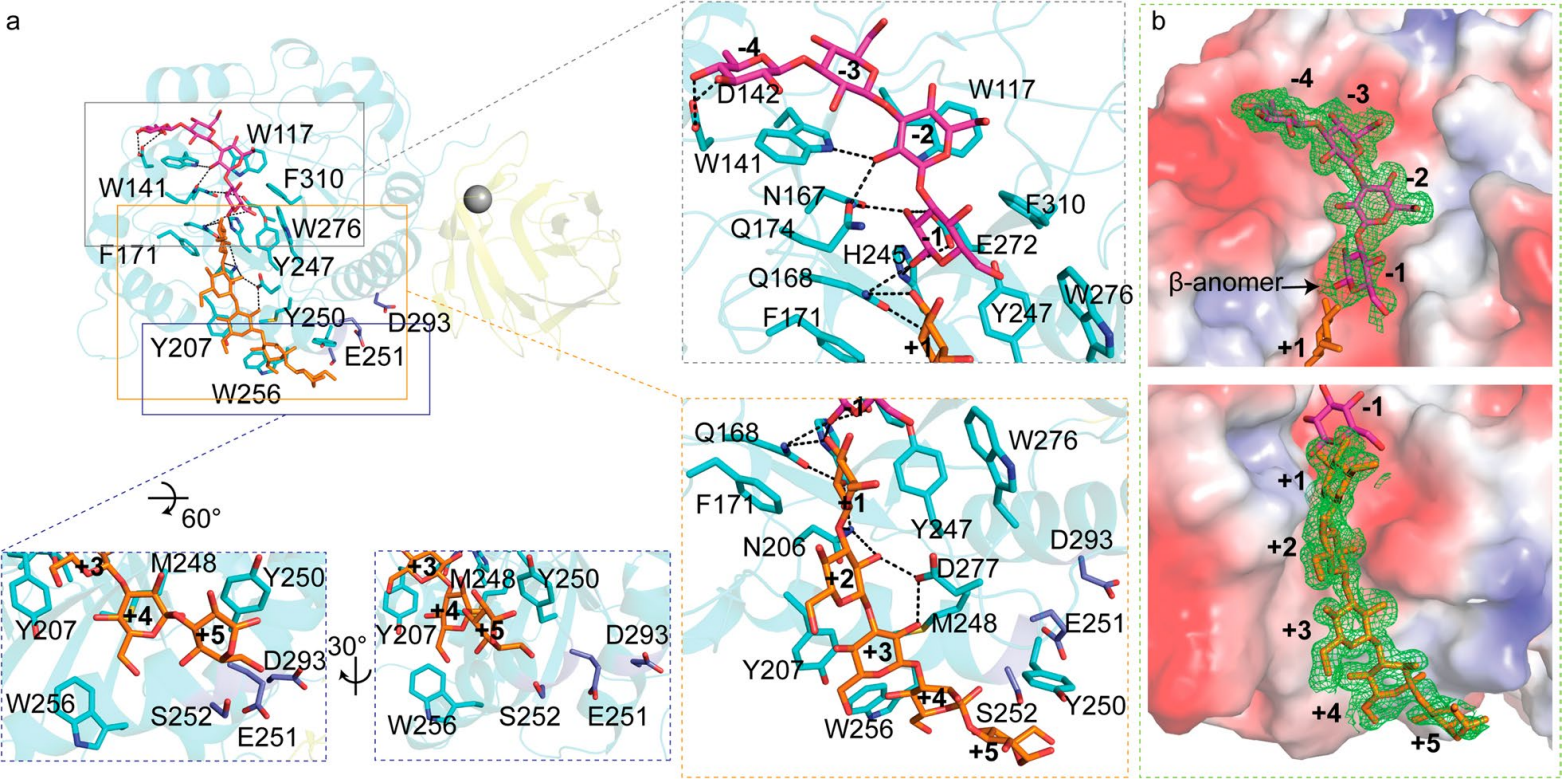
Substrate recognition within the GH128 catalytic domain of CBM6E. **a** Illustration of the interactions between active-site residues and β-1,3-glucooligosaccharides (GOS). GOS molecules with degrees of polymerization (DPs) of 4 and 5 are depicted in magenta and orange, respectively, representing their positions within the negative- and positive-subsite regions. The insets show expansions of the negative- and positive-subsite regions, as well as the region neighboring the + 5 subsite, respectively. Residues engaged in interactions with GOS are depicted as cyan-colored sticks, while residues neighboring the + 5 subsite, selected for tyrosine substitution, are portrayed as blue-colored sticks. **b** A polder map contoured at 2σ is presented for GOS with DPs of 4 and 5, observed within the negative and positive-subsite regions, respectively, as derived from the crystal structure of the E168Q inactive mutant of CBM6E-GH128/CBM6 cocrystallized with laminarihexaose. Additionally, CBM6E is represented using an electrostatic potential surface depiction

The crystal structure of CBM6E complexed with GOS was obtained through cocrystallization of the E168Q inactive mutant of CBM6E with laminarihexaose (L6) and determined at a high resolution of 1.28 Å. This complex structure enabled the mapping of two β-1,3-GOS chains within the catalytic groove of the GH128 domain: one spanning from the -4 to -1 subsites and the other extending from the + 1 to + 5 subsites (Fig. 2a). The cleavage of the glycosidic bond between the − 1 and + 1 subsites might be attributed to residual enzyme activity of the E168Q mutant enzyme at high crystallization concentrations. The elongated catalytic groove of CBM6E aptly accommodates the curved conformations of the two GOS chains, which collectively form a single-helical structure (Figs. 2b and 7c). This structural characteristic correlates with CBM6E's preference for linear curdlan over branched laminarin, as well as its distinctive cleavage pattern yielding products L3 to L6.

The interactions within the negative-subsite region are primarily governed by two conserved hydrophobic residues: W141, spanning from − 4 to − 2, and W117 at the − 2 subsite. At the − 4 subsite, D142 forms hydrogen bonds with the glucosyl C4-OH and C6-OH, occupying the position of water molecules observed at the − 5 and − 4 subsites in AmGH128_I, a member of the GH128 subgroups I (Additional file 1: Fig. S3a and b). The glucoside at the − 2 subsite forms hydrogen bonds with Q174. The glucoside at the − 1 subsite establishes hydrogen bonds with N167, Q168, H245, and E272. Additionally, F310 stacks against the glucosyl moiety at the − 1 subsite. Furthermore, a β-configuration is trapped within the − 1 subsite (Fig. 2b), which aligns with the retaining mechanism observed in the GH128 family. The conserved residue Y247 serves as a nucleophile-activating element, while E168 and E272 function as catalytic acid residues.

### Positive-subsite region

In the reported complex structures of subgroup II of the GH128 family, no glucosyl moieties were observed in the positive-subsite regions. We were fortunate to observe glucosyl moieties from + 1 to + 5 in the complex structure of CBM6E (Fig. 2a and c). The + 1 glucoside interacts with aromatic residues F171, Y247, and W276, which correspond to L117, Y192, and W218 of PvGH128_II, respectively (Additional file 1: Fig. S3d). Additionally, Q168 forms hydrogen bonds with the + 1 glucosyl C2-OH and C3-OH. N206 (corresponding to N152 of PvGH128_II) forms hydrogen bonds with the + 1 and + 2 glucosyl C2 hydroxyl groups, as well as the glycosidic bond between the + 1 and + 2 glucosides. D277 forms a hydrogen bond with the + 2 and + 3 glucosyl C2 hydroxyl groups. The + 3 glucoside stacks against Y207 and M248. Y207 corresponds to W153 in PvGH128_II and Y137 for the + 2 subsite in AmGH128_I (Additional file 1: Fig. S3c). Furthermore, W256 and Y250 (corresponding to S198 and Y194 of PvGH128_II, respectively) provide aromatic platforms for the + 4 and + 5 glucosides, respectively. These observations provide additional confirmation that CBM6E is classified within subgroup II of the GH128 family, given its stronger resemblance to PvGH128_II in the positive-subsite region compared to AmGH128_I.

### Rational design at the substrate-binding groove

To enhance the production of high-DP GOS, we pursued the extending of the substrate-binding groove by incorporating aromatic residues close to the + 5 subsite based on the CBM6E:GOS complex structure (Fig. 2a). While S252Y displayed approximately a twofold reduction in activity, with a specific activity of 1.9 U/mg compared to the wild-type (WT) protein’s (4.3 U/mg), both E251Y and D293Y exhibited approximately a threefold increase in activity toward curdlan high-set gel compared to the WT, with specific activities of 13.6 U/mg and 11.6 U/mg, respectively (Table 1).

**Table 1:** Specific activities of CBM6E WT and rational design variants using curdlan high-set gel at 15 mg/mL concentration as substrate

Protein name	Specific activity (U/mg)^a^
WT	4.3 ± 0.2^b^
E251Y	13.6 ± 0.4
S252Y	1.9 ± 0.1
D293Y	11.6 ± 0.5

^a^One unit of enzyme activity corresponds to 1 μmol of glucose released in 1 min. Measurements were conducted in triplicate for each enzyme at 35 °C, using a buffer solution consisting of 50 mM Na_2_HPO_2_–KH_2_PO_4_ at pH 6.0. The reported values represent the means accompanied by their respective standard deviations

^b^The specific activity of CBM6E WT is from the reference [19]

**Fig. 3:**
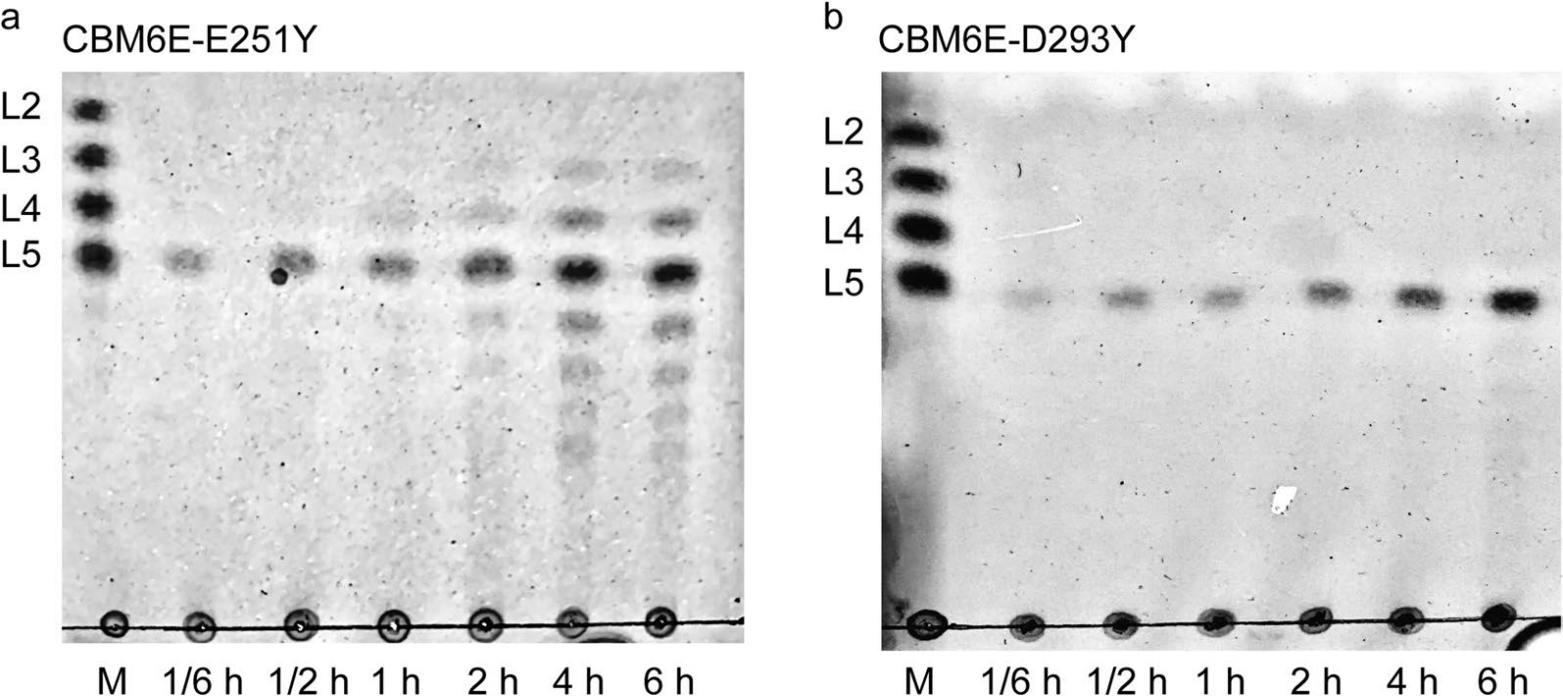
TLC patterns of hydrolytic products of curdlan high-set gel catalyzed by the E251Y (**a**) and D293Y (**b**) variants of CBM6E

Thin-layer chromatography (TLC) revealed that the E251Y variant generated a more diversified DP of GOS ranging from 3 to 9 (Fig. 3a). Conversely, the D293Y variant yielded relatively pure L5 from curdlan, as depicted by the results (Fig. 3b).

### Molecular docking study using a triple-helical β-1,3-glucan

**Fig. 4:**
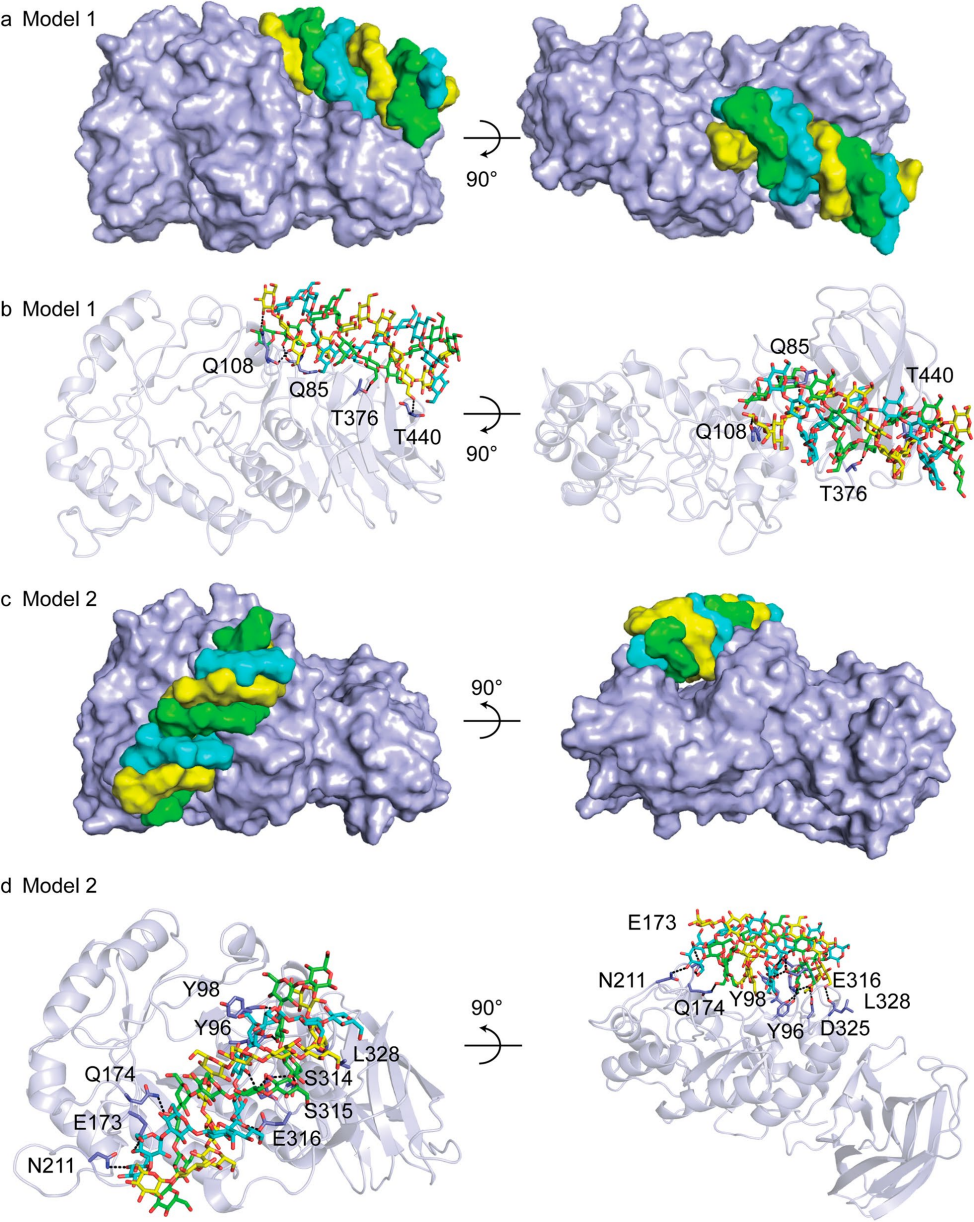
Two best-ranked docking poses of triple-helical curdlan to CBM6E. Surface complementarity (**a**, **c**) and hydrogen bonding interactions between triple-helical β-1,3-glucan and CBM6E (**b**, **d**) are indicated for both simulated structures

The enhanced specificity of CBM6E toward curdlan high-set gel compared to curdlan powder suggests that CBM6E has a preference for the triple-helical conformation of curdlan. To identify ancillary binding sites for curdlan triple helices, we conducted molecular docking experiments using a triple-helical β-1,3-glucan undecamer and the apo structure of CBM6E. Two binding models (Model 1 and Model 2) were generated and depicted in Fig. 4 and Additional file 1: Fig. S4, both exhibiting a ZDock Score of 11.7.

In Model 1, the triple-helical β-1,3-glucan displayed a favorable structural complementarity with the CBM6 domain, while its nonreducing ends made contact with the GH128 domain (Fig. 4a and Additional file 1: Fig. S4a). Notably, this model showed binding of the triple helix substrate to a site spanning both catalytic and CBM domains. In this model, the interaction between the triple-helical β-1,3-glucan and CBM6E was facilitated by the formation of six hydrogen bonds. These hydrogen bonds involved the main chain atoms of Q85, Q108, and T440, as well as the side chain atoms of Q108 and T376 (Fig. 4b). Additionally, hydrophobic interactions occurred between the glucan and CBM6E, mediated by residues S131, P353, M439, and S442 (Additional file 1: Fig. S4b).

Model 2 revealed the triple-helical β-1,3-glucan spanning across the active groove of the GH128 domain (Fig. 4c and Additional file 1: Fig. S4c). In this model, a total of fourteen hydrogen bonds were observed between the triple-helical β-1,3-glucan and CBM6E. These hydrogen bonds involved the main chain atoms of Y98, S314, and L328, as well as the side chain atoms of Y96, E173, Q174, N211, S315, E316, and D325 (Fig. 4d). Furthermore, hydrophobic interactions were identified between the glucan and CBM6E, with residues Y98, R313, and S315 playing a role in these interactions (Additional file 1: Fig. S4d).

Overall, both docking models, in conjunction with the crystal structure of CBM6E in complex with GOS, support the hypothesis that triple-helical curdlan unwinding precedes its hydrolysis within the catalytic groove.

### Mutational analysis of potential ancillary binding sites for triple-helical curdlan

To evaluate the functional roles of these potential ancillary binding sites in accommodating triple-helical curdlan, we introduced alanine substitutions for all surface-exposed residues (Y98, Q108, E173, S315, E316 in the GH128 domain, and T376 in the CBM6 domain) among them (Additional file 1: Fig. S5a) and subsequently conducted enzymatic kinetics assays. The results are summarized in Table 2 and illustrated in Additional file 1: Fig. S6.

**Table 2:** Kinetic parameters of CBM6E WT and variants targeting potential ancillary binding sites for triple-helical curdlan, as identified by molecular docking, using curdlan high-set gels as substrate

Protein name	*K*_0.5_^a^ (mg/mL)	*k*_cat_^a^ (s^−1^)	Hill coefficient (h)^a^	*k*_cat_/*K*_0.5_^a^ (s^−1^ mg^−1^ mL)
WT	7.6 ± 0.3	0.7 ± 0.05	2.5 ± 0.6	0.09
Y98A	7.7 ± 0.4	0.6 ± 0.03	1.9 ± 0.3	0.08
Q108A	8.5 ± 0.7	0.6 ± 0.05	1.6 ± 0.2	0.07
E173A	7.9 ± 0.5	0.6 ± 0.05	2.2 ± 0.5	0.08

	*K*_m_^b^ (mg/mL)	*k*_cat_^b^ (s^−1^)	/^b^	*k*_cat_/*K*_m_^b^ (s^−1^ mg^−1^ mL)
WT	16.7 ± 3.1	1.3 ± 0.13	/	0.08
S315A	20.4 ± 1.6	0.9 ± 0.05	/	0.04
E316A	18.2 ± 2.0	0.8 ± 0.05	/	0.04
T376A	24.1 ± 1.4	0.9 ± 0.03	/	0.04
S315A/T376A	N/A^c^, > 25	0.7 ± 0.09	/	N/A^c^
E316A/T376A	N/A^c^, > 25	0.8 ± 0.1	/	N/A^c^

^a^The data from the WT, Y98A, Q108A, and E173A variants were fitted well using the Hill model, as opposed to the Michaelis–Menten model

^b^The data from the S315A, E316A, T376A, S315A/T376A, and E316A/T376A mutants did not converge when using the Hill model; hence, a simple Michaelis–Menten model was employed instead. The WT data were re-fitted for comparison using the Michaelis–Menten model

^c^*K*_m_ values for the combined variants were not calculated due to insufficient saturation levels for accurate extrapolation

The data from the WT, Y98A, Q108A, and E173A variants were fitted well using the Hill model, as opposed to the Michaelis–Menten model. Notably, all Hill coefficients for both the WT and these mutant CBM6E enzymes exceeded 1, suggesting the existence of two or more binding sites and positive cooperativity in substrate binding. The Y98A, Q108A, and E173A variants displayed *K*_0.5_ values of 7.7, 8.5, and 7.9, respectively, which are comparable to the WT’s *K*_0.5_ value of 7.6 mg/mL, indicating minimal impact on substrate binding by these mutations.

In contrast, the data from the S315A, E316A, T376A, S315A/T376A, and E316A/T376A mutants did not converge when using the Hill model; hence, a simple Michaelis–Menten model was employed instead. For comparison, the WT data were re-fitted using the Michaelis–Menten model. The S315A and E316A variants displayed slight increases in *K*_m_ values, measured at 20.4 and 18.2 mg/mL, respectively, compared to the WT’s *K*_m_ value of 16.7 mg/mL. Conversely, the T376A variant exhibited an even higher increase in *K*_m_ value, reaching 24.1 mg/mL, indicating a reduced apparent affinity for the substrate due to this mutation. Furthermore, the *K*_m_ values for the combined variants S315A/T376A and E316A/T376A could not be determined due to saturation levels being too low for accurate extrapolation. Nevertheless, it is anticipated that these values are higher than the highest substrate concentrations (> 25 mg/mL), indicating substantially diminished affinities of these combined variants for curdlan high-set gels.

Collectively, these findings suggest that T376 within the CBM6 domain serves as a crucial ancillary binding site for triple-helical curdlan, while the GH128 and CBM6 domains work synergistically in recognizing curdlan.

### T376 in CBM6 represents a novel sugar-binding residue

Interestingly, the T376 residue in CBM6E-CBM6 is located outside the conventional sugar-binding clefts typically observed in CBM6 family members, as demonstrated by the structural alignment of CBM6E-CBM6 with ZgLamC_CBM6_ (Additional file 1: Fig. S5b). On the other hand, I382 and V435 in CBM6E-CBM6 correspond to Y291 and W348 in ZgLamC_CBM6_, respectively, which form the aromatic clamp in the variable loop site (VLS), previously referred to as cleft A. V388 in CBM6E-CBM6 corresponds to W297 in ZgLamC_CBM6_, a critical sugar-binding residue found in the concave face site (CFS), previously known as cleft B.

To assess the contribution of I382, V435, V388, and T376 to curdlan binding, we conducted pull-down assays and determined the dissociation constant (*K*_d_). The V435A mutant displayed a similar affinity for curdlan high-set gels as the WT, with a *K*_d_ of approximately 0.9 mg/mL. The T376A, V388A, and I382A variants of CBM6E-CBM6 exhibited approximately 27-, 21-, and 3-fold diminished binding affinity to curdlan high-set gels, with *K*_d_ values of 24.4, 18.8, and 3.0 mg/mL, respectively (Table 3 and Additional file 1: Fig. S7). A fluorescent thermal shift assay demonstrated that the T376A mutation had minimal impact on the observed melting temperature (*T*_m_) of CBM6E-GH128/CBM6, ruling out the possibility that the T376A mutation affects the overall folding and stability of CBM6E (Additional file 1: Fig. S8). These findings suggested that T376 in the CBM6 module represents a novel sugar-binding residue, which likely emerged during the coevolution of this CBM module with its appended GH module.

**Table 3:** Quantitative binding assay to curdlan high-set gel of CBM6E-CBM6 WT and its variants targeting potential sugar binding sites

Protein name	*K*_d_ (mg/mL)
CBM6E-CBM6-WT	0.9 ± 0.2
CBM6E-CBM6-V435A	0.9 ± 0.2
CBM6E-CBM6-I382A	3.0 ± 1.6
CBM6E-CBM6-T376A	24.4 ± 1.8
CBM6E-CBM6-V388A	18.8 ± 1.5

### CBM6 binding disturbed curdlan gel’s network structure

Certain CBMs have demonstrated the ability to disrupt the surface of tightly packed polysaccharides, such as cellulose fibers [26, 27] and starch granules [28, 29], leading to the loosening of the substrate and increased exposure to the catalytic module for enhanced degradation. To investigate whether the binding of CBM6E-CBM6 could induce disruption of curdlan gel’s ordered structures, both Congo red staining and SEM assays were performed.

In comparison to curdlan incubated with the negative control BSA, curdlan incubated with the CBM6E-CBM6 WT protein exhibited a notable hypsochromic shift from 497 to 492.5 nm, with a shifting distance of approximately 4.5 nm. This shift indicated a reduction in the ordered structures of curdlan. Conversely, curdlan treated with the binding-deficient mutant T376A of CBM6E-CBM6 exhibited no shift in comparison to curdlan incubated with BSA (Additional file 1: Fig. S9a). Crucially, this observed wavelength shift cannot be attributed to potential interaction disparities between Congo red and distinct proteins. This is substantiated by the absence of any shift in the absorption spectra when comparing Congo red incubated with both the WT and T376A mutant proteins, conducted without the addition of curdlan (Additional file 1: Fig. S9b).

**Fig. 5:**
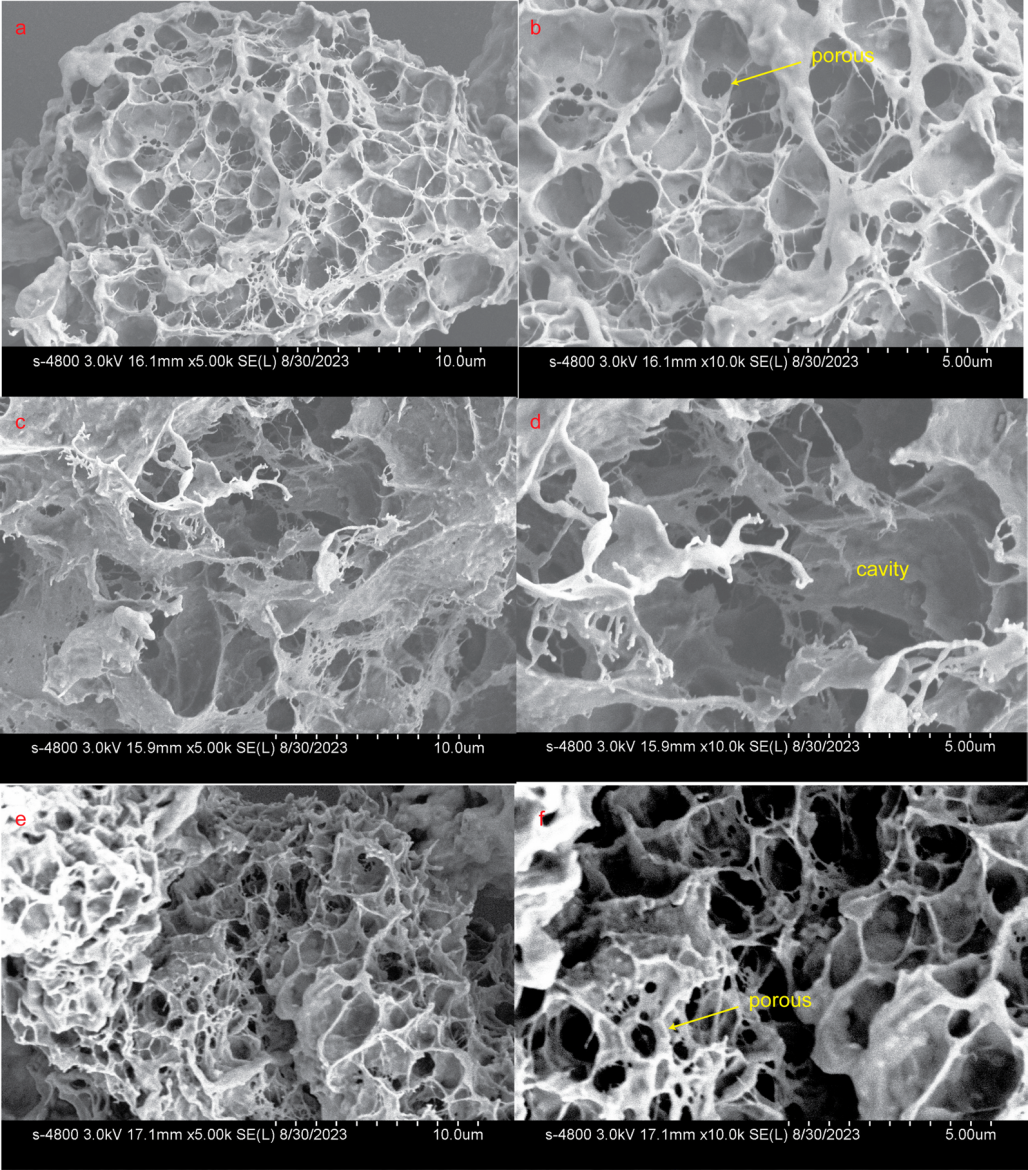
SEM visualization of microstructures in curdlan gel with various treatments. **a**, **b** Pure curdlan gel; **c**, **d** curdlan gel treated with CBM6E-CBM6-WT protein; **e**, **f** curdlan gel treated with the binding-deficient mutant protein, CBM6E-CBM6-T376A. The magnifications of each group images are set as 5000× and 10,000×

Moreover, the SEM images of the control sample containing pure curdlan gel revealed a consistent and tightly knit network structure with porous features (Fig. 5a and b). In contrast, after treatment with the CBM6E-CBM6 WT protein, noticeable disruptions emerged within the previously uniform curdlan gel structure, resulting in the formation of larger cavities (Fig. 5c and d). Conversely, the introduction of the binding-deficient mutant, CBM6E-CBM6-T376A, did not have a conspicuous impact on the network structure of the curdlan gel (Fig. 5e and f). These observations suggest that CBM6E-CBM6 effectively enhances the enzymatic hydrolysis of curdlan, not only by targeting but also by disruptive effects.

### Effect of CBM6 addition on enzymatic hydrolytic activity against curdlan

As the CBM6 module has the potential to disturb the compact structure of the curdlan gel, it may facilitate greater access for β-1,3-glucanases to the densely packed β-1,3-glucan chains. Consequently, this could lead to an enhancement in the activities of curdlan-degrading β-1,3-glucanases. In our investigation into the impact of CBM6 addition on curdlan hydrolysis, we specifically focused on two distinct β-1,3-glucanases: a GH128 enzyme, CBM6E, and a GH64 enzyme, KfGH64.

**Fig. 6:**
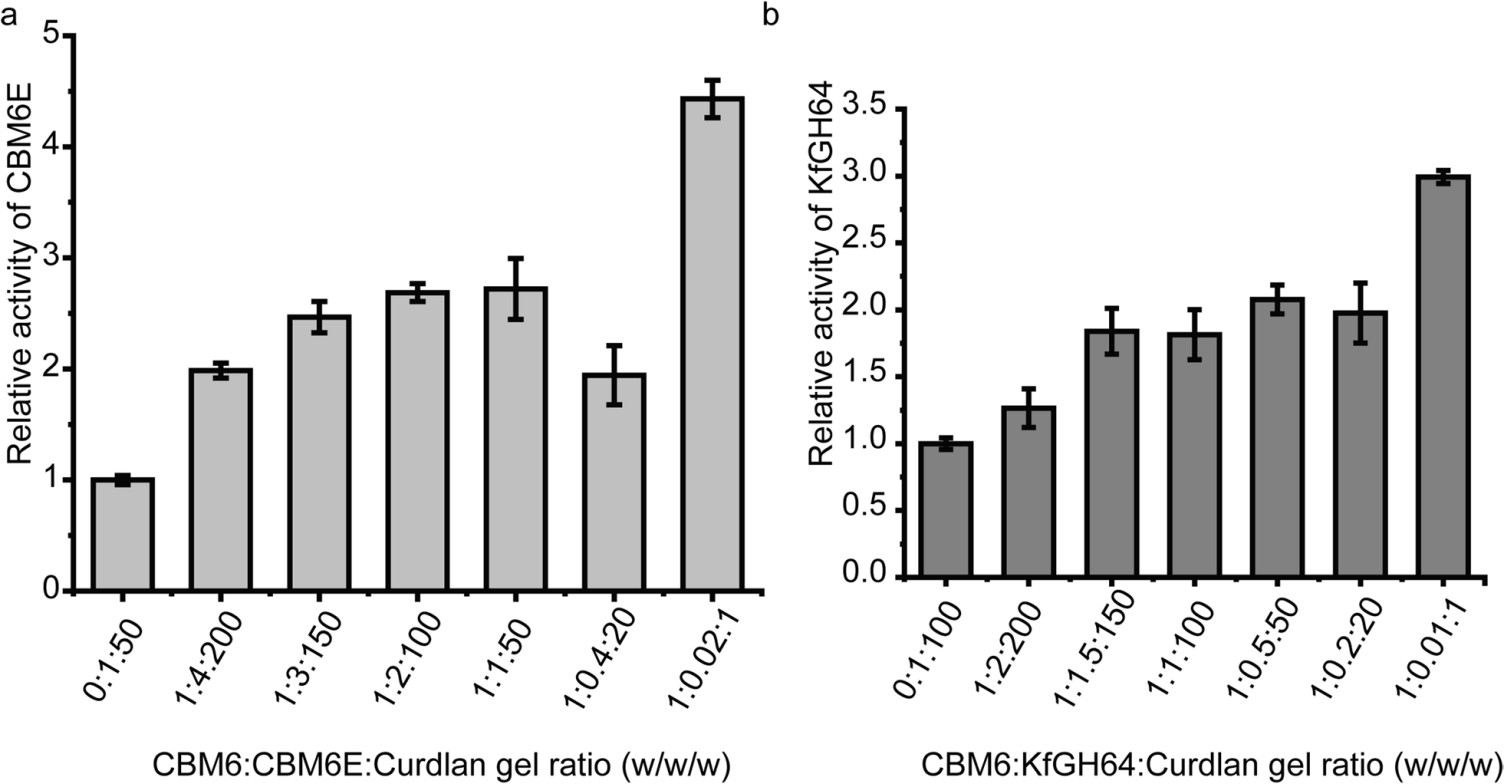
Increasing the CBM6 concentration during curdlan gel hydrolysis results in an increase in enzymatic activity. CBM6E-CBM6 was introduced in various ratios with curdlan gel (ranging from 1:200 to 1:1, w/w) before the enzyme was added into the mixture. The results are expressed as relative activities of CBM6E (**a**) and KfGH64 (**b**) against curdlan high-set gel in the absence and presence of CBM6E-CBM6. The consistent enzyme-to-curdlan gel mass ratios were maintained at 1:50 for CBM6E and gel, and 1:100 for KfGH64 and gel

When CBM6E-CBM6 was introduced in various ratios with curdlan gel (ranging from 1:200 to 1:20, and the respective ratios with the CBM6E enzyme varied from 1:4 to 2.5:1, w/w), it led to a remarkable two- to threefold increase in CBM6E activity compared to the negative control, where separate CBMs were absent. Particularly, at a CBM6:curdlan gel ratio of 1:1 (the corresponding ratio with the CBM6E enzyme was 50:1, w/w), the CBM6 incubation resulted in an approximately 4.5-fold increase in CBM6E activity (Fig. 6a).

Similarly, upon the addition of the CBM6 to the KfGH64-curdlan reaction mixture with different CBM6-to-curdlan gel ratios (ranging from 1:150 to 1:20 and the respective ratios with the KfGH64 enzyme varied from 1:1.5 to 5:1, w/w), we observed an approximate twofold increase in the rate of reducing sugar release. Notably, at a CBM6:curdlan gel ratio of 1:1 (the corresponding ratio with the KfGH64 enzyme was 100:1, w/w), an approximately threefold increase in the rate of reducing sugar release was achieved (Fig. 6b). These findings suggest that the promotive effect of the CBM6 on curdlan hydrolysis appears to be a consistent phenomenon across various β-1,3-glucanases.

## Discussion

**Fig. 7:**
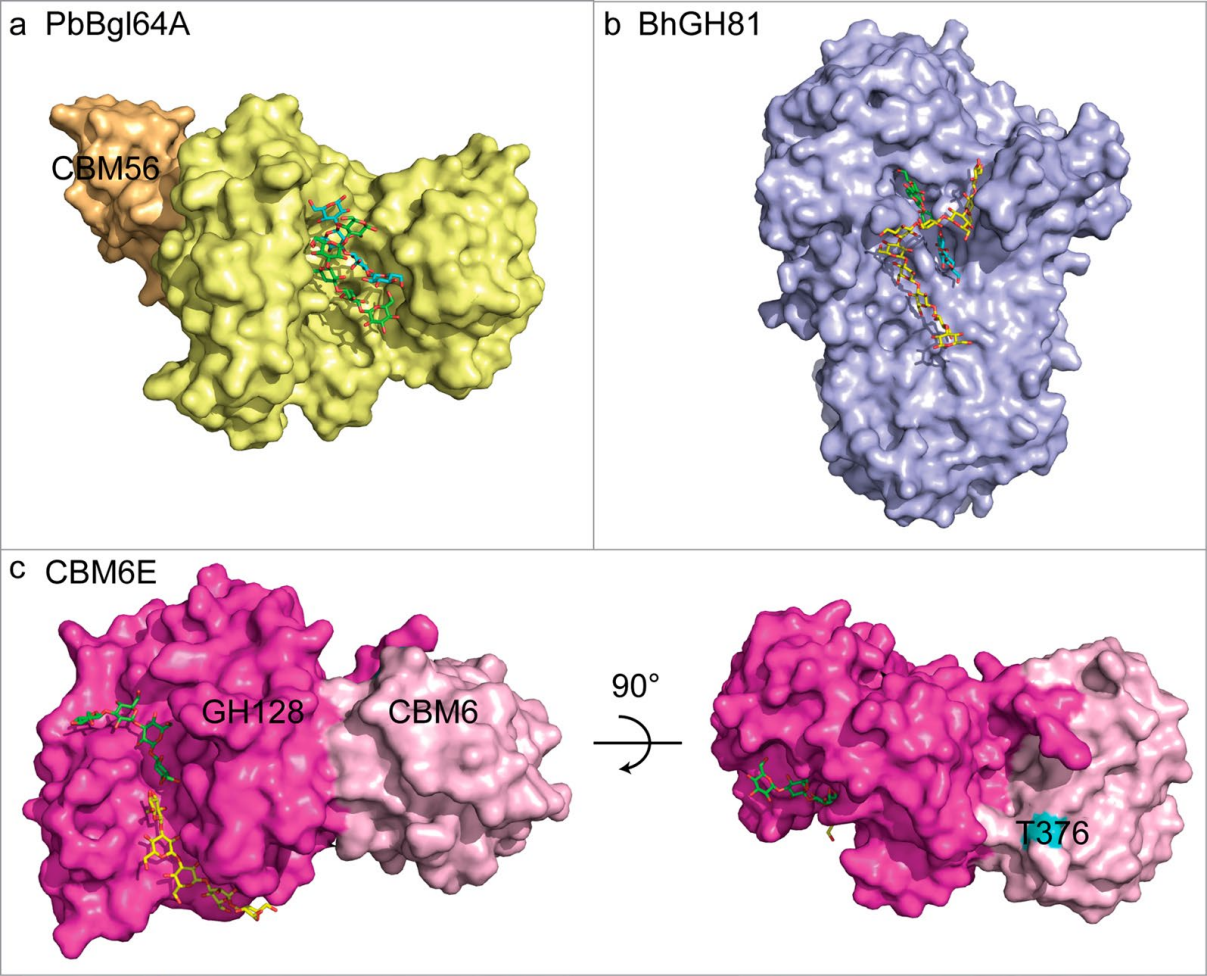
Crystal structures of endoglucanases bound to β-1,3-glucan chains. **a** Two β-1,3-glucooligosaccharide chains (DP = 4 and 5) bound to the active site of PbBgl64A (PDB ID: 5H9Y). **b** Three β-1,3-glucooligosaccharide chains (DP = 10, 3, and 2) bound to the active site of BhGH81 (PDB ID: 5T4G). **c** CBM6E bound to two β-1,3-glucooligosaccharide chains (DP = 4 and 5), with a novel and critical sugar-binding residue T376 within the CBM6 domain highlighted in blue. Proteins are shown in surface model and β-glucan chains in stick representation

This crystal structure of CBM6E in complex with GOS represents the first structure for the tandem GH128 and CBM6 module. Moreover, CEM6E is the third β-1,3-glucanase known to demonstrate curdlan hydrolytic activity while also furnishing comprehensive structural information. Structural comparative analyses among the three curdlan-degrading enzymes offer insights into the diverse strategies employed by enzymes to recognize the quaternary structures adopted by β-1,3-glucans. Some enzymes possess a broad and concave catalytic groove capable of directly accommodating triple- and/or double-helical β-1,3-glucans without encountering significant steric hindrance (Fig. 7a and b). In contrast, other enzymes feature a relatively narrower catalytic groove that exhibits a preference for binding single-helical β-1,3-glucan. This preference is often preceded by initial binding of triple-helical β-1,3-glucan to the ancillary binding regions of the enzyme, which subsequently unwinds the triple-helical structure into its single and double-helical forms (Fig. 7c).

The rational design applied to CBM6E presents an efficient approach for enhancing β-glucanase activity. This is achieved by extending the substrate-binding channel through the incorporation of aromatic amino acids at one or both ends. The elongated active-site tunnel, featuring Y293, is likely responsible for the creation of a new + 5 subsite, resulting in the D293Y mutant primarily producing L5 as its chief product. Moreover, Y251’s involvement in forming a new + 5 and/or + 6 subsite contributes to a broader product spectrum ranging from L3 to L9. These deliberate modifications in the interaction network between substrate and the enzyme’s active site are expected to elevate substrate binding affinity, thereby enhancing enzyme activity.

This study is also the first to report a CBM with the capacity to disrupt the organized gel structure of curdlan and subsequently enhance the activity of hydrolytic enzymes against curdlan. As we know, the primary function of a CBM is to bind to a carbohydrate ligand and target the catalytic machinery toward its substrate, thereby enhancing the catalytic efficiency of the multimodular carbohydrate-active enzyme. Another function of a CBM is to increase the enzyme’s concentration in close proximity to its substrate by recognizing not only substrate polysaccharides but also non-substrate polysaccharides [30]. A third, less commonly explored role of CBMs is the disruption of organized structures in recalcitrant substrates, rendering them more accessible to associated enzymes. While such a role has been documented for some cellulose binding [26, 27, 31, 32] and starch binding domains [28, 29], the supporting evidence remains limited. Here, we present an additional instance involving a β-1,3-glucan binding domain. The non-hydrolytic substrate disruption exhibited by these binding domains may result from the disruption of hydrogen bonds between saccharide chains. These modules are potential additives in the production of high-value oligosaccharides or fermentable sugars from recalcitrant insoluble polysaccharide substrates.

## Conclusions

This study delineates the structural characteristics and rational design of the GH128 and CBM6 tandem domain within CBM6E from a marine bacterium. The GH128 and CBM6 modules form a cohesive structural entity, jointly recognizing triple-helical β-1,3-glucans. Notably, CBM6 harbors an unconventional sugar-binding residue within the CBM6 family. The incorporation of hydrophobic residues at end subsites emerges as an effective strategy for extending the substrate-binding channel, enhancing the specific activity, and diversifying the GOS profile of endoglucanases. Additionally, this investigation identifies a non-hydrolytic yet disruptive CBM module targeting curdlan, serving as a beneficial addition to augment curdlan degradation by glucanases.

## Methods

### Cloning, expression, and purification

The gene encoding the GH128/CBM6 tandem module (residues 84–473) of CBM6E from *S. degradans* 2-40^T^ (GenBank accession number ABD80707.1) was chemically synthesized and cloned into the pET-22b vector. Using this construct as the PCR template, we amplified the genes encoding the GH128/CBM6 and CBM6 (residues 351–473) modules. These amplified genes were then cloned into a modified pET-28a vector, wherein the thrombin protease site was substituted with the tobacco etch virus (TEV) cleavage site. Subsequently, the plasmids were transformed into *Escherichia coli* BL21(DE3) cells for the production of recombinant proteins. All generated clones were verified by DNA sequencing. Expression of the proteins was induced by adding IPTG to a final concentration of 0.1 mM, followed by incubation of the cultures at 16 °C. Ni^2+^ affinity chromatography was employed to purify the proteins. Subsequently, the purified CBM6E-CBM6 protein was dialyzed into a buffer containing 20 mM Tris–HCl, pH 8.0, 200 mM NaCl.

To obtain the untagged CBM6E-GH128/CBM6 for crystallization, the His_6_-tag was removed through TEV protease digestion, followed by further purification using Ni^2+^ affinity chromatography. The wash fraction containing 20 mM imidazole was collected to remove the TEV protease carrying a His_6_-tag. Subsequent purification was carried out using a Superdex 200 HiLoad 16/60 column pre-equilibrated with a buffer containing 20 mM Tris–HCl, pH 8.0, and 500 mM NaCl.

For the preparation of the SeMet derivative protein, *E. coli* was cultured in 1 L of LR medium, which is a minimal medium described by LeMaster and Richards [33], at 37 °C until the absorbance at 600 nm reached approximately 0.8. Solid forms of 60 mg of SeMet and 50 mg each of threonine, lysine, phenylalanine, leucine, isoleucine, and valine were added to the culture. After further growth for 1 h at 16 °C, IPTG was added to a final concentration of 0.1 mM. Subsequent purification steps were carried out similarly to the native protein purification process.

To assess the overall structural stability, thermal shift assays were conducted for both CBM6E and its T376A mutant, following the methodology outlined in the referenced paper [34].

### Enzyme activity assays

Enzymatic specific activity, kinetics and TLC were all performed as previously described [19]. Briefly, enzymatic activity assays were performed at 35 °C in 50 mM Na_2_HPO_2_–KH_2_PO_4_ buffer (pH 6.0) using curdlan high-set gel as substrate, in triplicate. Reactions were initiated by adding 0.05 mL of enzyme solution (WT: 2.6 mg/mL, single-point mutant: 3.5 mg/mL, multiple-point mutant: 7 mg/mL) to 0.55 mL of curdlan gel, and reducing sugars were quantified using the DNS method. Nonlinear regression analysis with Origin software was used to fit Michaelis–Menten or Hill equations to the initial velocity data against substrate concentrations.

### Curdlan binding assays

Curdlan high-set gels with varying curdlan concentrations were prepared by heating 0.2 mL aliquots at 80 °C for 4 h, followed by gradual cooling to room temperature. Subsequently, WT or mutant CBM6E-CBM6 forms, or BSA (0.1 mL of 1 mg/mL), were added to the gels, which were then agitated at 4 °C for 1 h. Supernatants were analyzed for protein content, and a relative dilution factor was calculated based on free BSA recovery to adjust for wet curdlan dilution. Binding assays, performed in triplicate, measured bound protein as a fraction of total protein plotted against curdlan concentration. Data analysis utilized Origin software for nonlinear regression fitting to the total binding equation,

$$Y = B_{{\text{max}}} \times X/\left( {K_{\text{d}} + X} \right) + {\text{NS}} \times X + {\text{Background}},$$

where *Y* represents fraction of bound protein, *X* represents substrate amount, *K*_d_ represents dissociation constant, NS represents nonspecific binding slope, and Background denotes nonspecific binding in absence of ligand. *B*_max_ was constrained to 1.0 during curve fitting to reflect maximum binding fraction.

### Protein crystallization, X-ray data collection, and structure determination

Crystals were grown using the sitting drop vapor diffusion method. Specifically, 1 μL of protein solution was mixed with 1 μL of reservoir solution at 10 °C. Crystals of SeMet derivative CBM6E-GH128/CBM6 were obtained by crystallizing the protein using reservoir buffer 1, which consisted of 0.2 M ammonium sulfate, 0.1 M bis–tris at pH 5.5, and 25% (w/v) polyethylene glycol 3350. For the crystallization of CBM6E-GH128/CBM6 in complex with GOS, the catalytically deficient E168Q mutant of CBM6E-GH128/CBM6 was cocrystallized with L6 at a molar ratio of 1:3. This complex was crystallized using reservoir buffer 2, which contained 0.2 M magnesium chloride, 0.1 M bis–tris at pH 6.5, and 25% (w/v) polyethylene glycol 3350.

X-ray diffraction data were collected at 100 K using the BL19U1 beamline at the Shanghai Synchrotron Radiation Facility in China. Prior to data collection, crystals were cryoprotected using a reservoir solution supplemented with 25% glycerol. Data processing was carried out using XDS [35], and scaling was performed using AIMLESS, which is part of the CCP4i software suite [36]. The structure of the apo CBM6E-GH128/CBM6 was determined using the Single-wavelength Anomalous Diffraction (SAD) method with a SeMet derivative. Selenium sites were located using Shelx C/D software [37], and phase calculation and improvement were conducted using PHENIX.Autosol. An initial model was automatically built using PHENIX.AutoBuild [38]. For the complex structure of CBM6E-GH128/CBM6 with GOS, molecular replacement was employed in PHASER [39], using the apo CBM6E structure as the search model. All structures were refined using PHENIX.refine and Coot. Detailed data collection and refinement statistics are shown in Additional file 1: Table S1.

### Molecular docking

Molecular docking was performed using the ZDOCK protocol within BIOVIA Discovery Studio V17 software. The ligand used in the docking simulations was the triple-helical form of a β-1,3-glucan undecamer, consisting of eleven glucose residues. The receptor in the docking study was the crystal structure of apo CBM6E-GH128/CBM6. The ZDOCK algorithm [40] was employed for rigid body docking, utilizing shape complementarity, electrostatics, and desolvation terms to generate ZDock scores. These scores were used to rank the protein poses obtained from the docking simulations.

The best-ranked docking poses of the triple-helical β-1,3-glucan were further analyzed using PyMol and LigPlot. These tools were used to identify and study the hydrogen bonding and hydrophobic interactions between the ligand and the receptor protein.

### Congo red staining

Curdlan high-set gel was prepared by preheating it at 80 °C for 4 h. Each curdlan gel solution (250 μL of 0.05 mg/mL) was mixed with either the WT or T376A mutant of CBM6E-CBM6 or BSA protein (50 μL of 1 mg/mL), respectively. The mixture was incubated at 30 °C for 30 min, followed by staining with Congo red dye (250 μL of 0.1 mg/mL). Congo red was purchased from Shanghai Sangon Biotech Co., Ltd., China. To exclude the potential influence of Congo red's interaction with different proteins on absorption wavelength, we compared the spectra of Congo red when mixed with WT and mutant CBM6E-CBM6 proteins at equivalent concentrations without the addition of curdlan.

To obtain the absorption spectrum of the final solution, a UV–Vis spectrophotometer was used to scan the solution over a wavelength range of 400–650 nm at 1 nm intervals. All Congo red staining experiments were performed in triplicate, and the average values were used to generate the plots for analysis and comparison.

### SEM

Microstructural alterations in the curdlan gel following incubation with CBM6E-CBM6 were examined using SEM. Curdlan high-set gel was prepared by preheating it at 80 °C for 4 h. In the experimental group, 800 μL of a 20 mg/mL curdlan gel was incubated with 200 μL of either a 0.5 mg/mL CBM6E-CBM6 or CBM6E-CBM6-T376A mutant protein solution at 18 °C for 9 h. Conversely, the control group of pure curdlan gel underwent incubation with only a buffer (20 mM Tris–HCl, pH 8.0, 200 mM NaCl).

The incubated samples were snap-frozen and placed in an ultra-low temperature freezer at − 80 °C for 2 h, and then freeze-dried with a freeze dryer at − 40 °C for 12 h to obtain SEM samples. Before observation, the gel with gold was sprayed using HITACHI E-1010 ion sputter instrument.

### Enzymatic hydrolysis of curdlan with the CBM6 addition

The genetic sequence encoding a GH64 family β-1,3-glucanase, known as *kfgh64* (GenBank accession number ADB34580.1), was synthesized chemically and subsequently integrated into the pET-28a vector. The KfGH64 protein was synthesized in BL21(DE3) cells, induced by the addition of 0.1 mM IPTG, and cultivated at 16 °C. For protein purification, Ni^2+^ affinity chromatography was employed, followed by dialysis into a solution containing 20 mM Tris–HCl, pH 7.5, and 500 mM NaCl.

Curdlan high-set gel was prepared by preheating it at 80 °C for 4 h and used as the substrate. A 100 μL aliquot of the gel, with a concentration of 10 mg/mL, was incubated with varying concentrations of the CBM6E-CBM6 protein (100 μL) at 30 °C for 0.5 h. Subsequently, the reaction was initiated by introducing the KfGH64 enzyme (10 μL of 1 mg/mL) into the mixture at 37 °C for 5 min, using a solution containing 50 mM sodium acetate at pH 5.5. Alternatively, the CBM6E enzyme was added to the mixture (20 μL of 1 mg/mL) to initiate the reaction at 35 °C for 5 min in a Na_2_HPO4–KH_2_PO4 buffer with a pH of 6.0.

### Supplementary Information

**Additional file 1: Table S1**. Data collection and refinement statistics. **Figure S1**. Sequence conservation analysis of the CBM6E-GH128/CBM6 protein using the ConSurf web server. **Figure S2**. (a) Superimposition of CBM6E (gray) with ZgLamC_CBM6_ (PDB code 5fui, yellow). Residues coordinating a Mg^2+^ ion (green) within CBM6E-CBM6 and ZgLamC_CBM6_ are labeled in blue and orange, respectively. A Mg^2+^-coordinated water molecule is depicted as a cyan sphere. (b) Mg^2+^ coordination in CBM6E-CBM6 is illustrated by yellow full lines. (c) Modeling a Ca^2+^ ion into the same site resulted in a negative (red) Fo–Fc map for the ion after crystal structure refinement. **Figure S3**. (a) Structural superposition of AmGH128_I (PDB code 6UAS, blue) and CBM6E (green) with L5 (gray) and L4 (magenta) bound at the negative-subsite region, respectively. (b) Structural superposition of PvGH128_II (PDB code 6UAW, orange) and CBM6E (green) with L3 (gray) and L4 (magenta) bound at the negative-subsite region, respectively. (c) Structural superposition of AmGH128_I (PDB code 6UAU, blue) and CBM6E (green) with L2 (gray) and L5 (magenta) bound at the positive-subsite region, respectively. (d) Structural superposition of PvGH128_II (PDB code 6UAW, orange) and CBM6E (green) with no oligosaccharide and L5 (magenta) bound at the positive-subsite region, respectively. **Figure S4.** Structure superposition of CBM6E with oligosaccharides in the complex crystal structure and with a docked triple-helical β-1,3-glucan (a, c). Hydrophobic interactions between docked triple-helical β-1,3-glucans and CBM6E (b, d). **Figure S5**. (a) Electrostatic potential surface representation of CBM6E highlighting the exposed ancillary-binding site candidates for the anchoring of curdlan according to molecular docking of triple-helical β-1,3-glucoundecamers. (b) The superimposition of CBM6E-CBM6 (gray) with ZgLamC_CBM6_ (yellow). I382 and V435 of CBM6E correspond to Y291 and W348 of ZgLamC_CBM6_, the aromatic clamp in the variable loop site (VLS) where a glycerol (GOL) is found. V388 of CBM6E corresponds to W297 of ZgLamC_CBM6_, a key sugar-binding residue in the concave face site (CFS) where a 2-aminomethyl pyridine (APY) is found. T376 of CBM6E-CBM6 is not conserved in ZgLamC_CBM6_. Residues of CBM6E-CBM6 and ZgLamC_CBM6_ are labeled in blue and orange, respectively. **Figure S6**. (a-d) Fitted kinetic curves of CBM6E WT, Y98A, Q108A, and E173A variants using the Hill model. (e-j) Fitted kinetic curves of CBM6E WT, S315A, E316A, T376A, S315A/T376A, and E316A/T376A variants using the Michaelis–Menten model. **Figure S7**. (a-e) Fractions of CBM6E-CBM6 WT and its various mutants bound versus different amounts of curdlan high-set gels were plotted and fit. **Figure S8.** (a) Melting curves of CBM6E-GH128/CBM6 WT and its T376A mutant. (b) The first derivatives of the melting curves of CBM6E-GH128/CBM6 WT and its T376A mutant showing T_m_ values of 38.8 ℃ and 38.3 ℃, respectively. **Figure S9.** Absorption spectra of the Congo red-curdlan complexes. (a) Curdlan was incubated with CBM6E-CBM6, the T376A mutant of CBM6E-CBM6 and BSA, respectively. After staining the samples with Congo red dye, the absorption spectra were recorded and plotted. (b) Absorbance of Congo red after incubation with the WT and T376A mutant of CBM6E-CBM6 at the same concentration without the addition of curdlan.

## Data Availability

The atomic coordinates for apo CBM6E and CBM6E-E168Q complexed with oligosaccharides have been deposited in the RCSB Protein Data Bank with accession numbers 8J3X and 8J3Y. The authors declare that the data supporting the findings of this study are available within the paper and its additional Information files. Should any raw data files be needed in another format, they are available from the corresponding author upon reasonable request.

## References

[1] Zhang R, Edgar KJ (2014). Properties, chemistry, and applications of the bioactive polysaccharide curdlan. Biomacromol.

[2] Chaudhari V, Buttar HS, Bagwe-Parab S, Tuli HS, Vora A, Kaur G (2021). Therapeutic and industrial applications of curdlan with overview on its recent patents. Front Nutr.

[3] Tang J, Zhen H, Wang N, Yan Q, Jing H, Jiang Z (2019). Curdlan oligosaccharides having higher immunostimulatory activity than curdlan in mice treated with cyclophosphamide. Carbohyd Polym.

[4] Shim JH, Sung KJ, Cho MC, Choi WA, Yang Y, Lim JS, Yoon DY (2007). Antitumor effect of soluble beta-1,3-glucan from *Agrobacterium* sp. R259 KCTC 1019. J Microbiol Biotechnol.

[5] Wan-Mohtar WA, Young L, Abbott GM, Clements C, Harvey LM, McNeil B (2016). Antimicrobial properties and cytotoxicity of sulfated (1,3)-β-D-glucan from the mycelium of the mushroom *Ganoderma lucidum*. J Microbiol Biotechnol.

[6] Klarzynski O, Plesse B, Joubert JM, Yvin JC, Kopp M, Kloareg B, Fritig B (2000). Linear beta-1,3 glucans are elicitors of defense responses in tobacco. Plant Physiol.

[7] Kumar K, Rajulapati V, Goyal A (2020). In vitro prebiotic potential, digestibility and biocompatibility properties of laminari-oligosaccharides produced from curdlan by β-1,3-endoglucanase from *Clostridium thermocellum*. 3 Biotech.

[8] Shi Y, Liu J, Yan Q, You X, Yang S, Jiang Z (2018). In vitro digestibility and prebiotic potential of curdlan (1–>3)-beta-d-glucan oligosaccharides in Lactobacillus species. Carbohyd Polym.

[9] Adams EL, Rice PJ, Graves B, Ensley HE, Yu H, Brown GD, Gordon S, Monteiro MA, Papp-Szabo E, Lowman DW (2008). Differential high-affinity interaction of dectin-1 with natural or synthetic glucans is dependent upon primary structure and is influenced by polymer chain length and side-chain branching. J Pharmacol Exp Ther.

[10] Fu YB, Cheng LK, Meng YY, Li SG, Zhao XM, Du YG, Yin H (2015). Cellulosimicrobium cellulans strain E4–5 enzymatic hydrolysis of curdlan for production of (1 -> 3)-linked beta-D-glucan oligosaccharides. Carbohyd Polym.

[11] Kumagai Y, Okuyama M, Kimura A (2016). Heat treatment of curdlan enhances the enzymatic production of biologically active beta-(1,3)-glucan oligosaccharides. Carbohyd Polym.

[12] Li KK, Chen W, Wang WX, Tan HD, Li SG, Yin H (2018). Effective degradation of curdlan powder by a novel endo-beta-1 -> 3-glucanase. Carbohyd Polym.

[13] Li J, Zhu L, Zhan X-B, Xu M, Lin C-C, Zheng Z-Y, Li W-J (2014). Purification and characterization of a new endo-β-1,3-glucanase exhibiting a high specificity for curdlan for production of β-1,3-glucan oligosaccharides. Food Sci Biotechnol.

[14] Yi P, Yan Q, Jiang Z, Wang L (2018). A first glycoside hydrolase family 50 endo-beta-1,3-d-glucanase from *Pseudomonas aeruginosa*. Enzyme Microb Technol.

[15] Helbert W, Poulet L, Drouillard S, Mathieu S, Loiodice M, Couturier M, Lombard V, Terrapon N, Turchetto J, Vincentelli R (2019). Discovery of novel carbohydrate-active enzymes through the rational exploration of the protein sequences space. Proc Natl Acad Sci U S A.

[16] Pluvinage B, Fillo A, Massel P, Boraston AB (2017). Structural analysis of a family 81 glycoside hydrolase implicates its recognition of beta-1,3-Glucan quaternary structure. Structure.

[17] Qin Z, Yang D, You X, Liu Y, Hu S, Yan Q, Yang S, Jiang Z (2017). The recognition mechanism of triple-helical beta-1,3-glucan by a beta-1,3-glucanase. Chem Commun (Camb).

[18] Yu PX, Zhou F, Yang D (2019). Curdlan conformation change during its hydrolysis by multi-domain beta-1,3-glucanases. Food Chem.

[19] Jia X, Wang C, Du X, Peng H, Liu L, Xiao Y, He C (2021). Specific hydrolysis of curdlan with a novel glycoside hydrolase family 128 β-1,3-endoglucanase containing a carbohydrate-binding module. Carbohyd Polym.

[20] Santos CR, Costa P, Vieira PS, Gonzalez SET, Correa TLR, Lima EA, Mandelli F, Pirolla RAS, Domingues MN, Cabral L (2020). Structural insights into β-1,3-glucan cleavage by a glycoside hydrolase family. Nat Chem Biol.

[21] Jam M, Ficko-Blean E, Labourel A, Larocque R, Czjzek M, Michel G (2016). Unraveling the multivalent binding of a marine family 6 carbohydrate-binding module with its native laminarin ligand. FEBS J.

[22] Czjzek M, Ben David A, Bravman T, Shoham G, Henrissat B, Shoham Y (2005). Enzyme-substrate complex structures of a GH39 beta-xylosidase from Geobacillus stearothermophilus. J Mol Biol.

[23] Yu S, Su T, Wu H, Liu S, Wang D, Zhao T, Jin Z, Du W, Zhu MJ, Chua SL (2015). PslG, a self-produced glycosyl hydrolase, triggers biofilm disassembly by disrupting exopolysaccharide matrix. Cell Res.

[24] Ficko-Blean E, Boraston AB (2012). Insights into the recognition of the human glycome by microbial carbohydrate-binding modules. Curr Opin Struct Biol.

[25] Hettle A, Fillo A, Abe K, Massel P, Pluvinage B, Langelaan DN, Smith SP, Boraston AB (2017). Properties of a family 56 carbohydrate-binding module and its role in the recognition and hydrolysis of beta-1,3-glucan. J Biol Chem.

[26] Bernardes A, Pellegrini VOA, Curtolo F, Camilo CM, Mello BL, Johns MA, Scott JL, Guimaraes FEC, Polikarpov I (2019). Carbohydrate binding modules enhance cellulose enzymatic hydrolysis by increasing access of cellulases to the substrate. Carbohyd Polym.

[27] Gao PJ, Chen GJ, Wang TH, Zhang YS, Liu J (2001). Non-hydrolytic disruption of crystalline structure of cellulose by cellulose binding domain and linker sequence of cellobiohydrolase I from *Penicillium janthinellum*. Sheng wu hua xue yu sheng wu wu li xue bao Acta biochimica et biophysica Sinica.

[28] Southall SM, Simpson PJ, Gilbert HJ, Williamson G, Williamson MP (1999). The starch-binding domain from glucoamylase disrupts the structure of starch. FEBS Lett.

[29] Peng H, Li R, Li F, Zhai L, Zhang X, Xiao Y, Gao Y (2018). Extensive hydrolysis of raw rice starch by a chimeric α-amylase engineered with α-amylase (AmyP) and a starch-binding domain from *Cryptococcus* sp. S-2. Appl Microbiol Biotechnol.

[30] Hervé C, Rogowski A, Blake AW, Marcus SE, Gilbert HJ, Knox JP (2010). Carbohydrate-binding modules promote the enzymatic deconstruction of intact plant cell walls by targeting and proximity effects. Proc Natl Acad Sci U S A.

[31] Quiroz-Castañeda RE, Martínez-Anaya C, Cuervo-Soto LI, Segovia L, Folch-Mallol JL (2011). Loosenin, a novel protein with cellulose-disrupting activity from Bjerkandera adusta. Microb Cell Fact.

[32] Wang Y, Tang R, Tao J, Gao G, Wang X, Mu Y, Feng Y (2011). Quantitative investigation of non-hydrolytic disruptive activity on crystalline cellulose and application to recombinant swollenin. Appl Microbiol Biotechnol.

[33] LeMaster DM, Richards FM (1982). Preparative-scale isolation of isotopically labeled amino acids. Anal Biochem.

[34] Du X, Chu X, Liu N, Jia X, Peng H, Xiao Y, Liu L, Yu H, Li F, He C (2023). Structures of the NDP-pyranose mutase belonging to glycosyltransferase family 75 reveal residues important for Mn(2+) coordination and substrate binding. J Biol Chem.

[35] Kabsch WXDS (2010). Xds. Acta Crystallogr D Biol Crystallogr.

[36] Winn MD, Ballard CC, Cowtan KD, Dodson EJ, Emsley P, Evans PR, Keegan RM, Krissinel EB, Leslie AG, McCoy A (2011). Overview of the CCP4 suite and current developments. Acta Crystallogr D Biol Crystallogr.

[37] Sheldrick GM (2008). A short history of SHELX. Acta Crystallogr A.

[38] Adams PD, Afonine PV, Bunkoczi G, Chen VB, Davis IW, Echols N, Headd JJ, Hung LW, Kapral GJ, Grosse-Kunstleve RW (2010). PHENIX: a comprehensive Python-based system for macromolecular structure solution. Acta Crystallogr D Biol Crystallogr.

[39] McCoy AJ, Grosse-Kunstleve RW, Adams PD, Winn MD, Storoni LC, Read RJ (2007). Phaser crystallographic software. J Appl Crystallogr.

[40] Chen R, Weng Z (2002). Docking unbound proteins using shape complementarity, desolvation, and electrostatics. Proteins.

