# Supplementary material for: Structural insights into curdlan degradation via a glycoside hydrolase containing a disruptive carbohydrate-binding module
Source: Biotechnol Biofuels Bioprod. 2024 Mar 21;17:45. doi: 10.1186/s13068-024-02494-5 (PMC10956234; doi:10.1186/s13068-024-02494-5)
Supplement: Supplementary file 1 — Additional file 1: Table S1. Data collection and refinement statistics. Figure S1. Sequence conservation analysis of the CBM6E-GH128/CBM6 protein using the ConSurf web server. Figure S2. (a) Superimposition of CBM6E (gray) with ZgLamCCBM6 (PDB code 5fui, yellow). Residues coordinating a Mg2+ ion (green) within CBM6E-CBM6 and ZgLamCCBM6 are labeled in blue and orange, respectively. A Mg2+-coordinated water molecule is depicted as a cyan sphere. (b) Mg2+ coordination in CBM6E-CBM6 is illustrated by yellow full lines. (c) Modeling a Ca2+ ion into the same site resulted in a negative (red) Fo–Fc map for the ion after crystal structure refinement. Figure S3. (a) Structural superposition of AmGH128_I (PDB code 6UAS, blue) and CBM6E (green) with L5 (gray) and L4 (magenta) bound at the negative-subsite region, respectively. (b) Structural superposition of PvGH128_II (PDB code 6UAW, orange) and CBM6E (green) with L3 (gray) and L4 (magenta) bound at the negative-subsite region, respectively. (c) Structural superposition of AmGH128_I (PDB code 6UAU, blue) and CBM6E (green) with L2 (gray) and L5 (magenta) bound at the positive-subsite region, respectively. (d) Structural superposition of PvGH128_II (PDB code 6UAW, orange) and CBM6E (green) with no oligosaccharide and L5 (magenta) bound at the positive-subsite region, respectively. Figure S4. Structure superposition of CBM6E with oligosaccharides in the complex crystal structure and with a docked triple-helical β-1,3-glucan (a, c). Hydrophobic interactions between docked triple-helical β-1,3-glucans and CBM6E (b, d). Figure S5. (a) Electrostatic potential surface representation of CBM6E highlighting the exposed ancillary-binding site candidates for the anchoring of curdlan according to molecular docking of triple-helical β-1,3-glucoundecamers. (b) The superimposition of CBM6E-CBM6 (gray) with ZgLamCCBM6 (yellow). I382 and V435 of CBM6E correspond to Y291 and W348 of ZgLamCCBM6, the aromatic clamp in the variable loop site (VLS) [file 13068_2024_2494_MOESM1_ESM.docx]

Journal name: Biotechnology for Biofuels and Bioproducts

**Structural insights into curdlan degradation via a glycoside hydrolase containing a disruptive carbohydrate binding module**

Tianhang Lv^1#^, Juanjuan Feng^1#^, Xiaoyu Jia^1#^, Cheng Wang^1^, Fudong Li^2^, Hui Peng^1^, Yazhong Xiao^1^, Lin Liu^1^, Chao He^1*^

^1^School of Life Sciences and Anhui Key Laboratory of Modern Biomanufacturing, Anhui University, Hefei, Anhui, China;

^2^MOE Key Laboratory for Cellular Dynamics, School of Life Sciences, Division of Life Sciences and Medicine, University of Science and Technology of China, Hefei,

China

^#^These authors contributed equally to this work.

^*^**Corresponding author**: Chao He, [chaohe@ahu.edu.cn](mailto:chaohe@ahu.edu.cn)

**Table S1**. Data collection and refinement statistics.

|  | CBM6E apo | CBM6E E168Q in complex with oligosaccharides |
| --- | --- | --- |
| **Data Collection** |  |  |
| Wavelength(Å) | 0.9792 | 0.9792 |
| Space group | *P*2_1_2_1_2 | *P*2_1_ |
| Cell parameters |  |  |
| a, b, c (Å) | 84.81, 146.03, 76.85 | 63.48, 97.80, 72.42 |
| α, β, γ (°) | 90, 90, 90 | 90, 108.4, 90 |
| Resolution(Å) | 19.68-2.40  (2.49-2.40)^a^ | 40.00-1.28  (1.30-1.28) |
| *R*_merge_ (%) | 13.1 (93.1) | 6.8 (75.9) |
| *CC*_1/2_(%) | 99.6 (99.2) | 99.7 (75.7) |
| *I*/σ*I* | 10.7 (1.9) | 25.0 (2.2) |
| Completeness (%) | 99.5 (99.2) | 100 (100) |
| Average redundancy | 8.0 (8.3) | 6.6 (6.4) |
|  |  |  |
| **Refinement** |  |  |
| No. reflections (overall) | 37767 | 204336 |
| No. reflections (test set) | 1836 | 10095 |
| *R*_work_/*R*_free_(%) | 18.24/23.27 | 14.94/16.81 |
| Number of atoms |  |  |
| Protein/Sugars | 6257/- | 6402/204 |
| H_2_O/Mg^2+^ | 174/2 | 1332/2 |
| B factors (Å^2^) |  |  |
| Protein/Sugars | 52.44/- | 16.01/33.88 |
| H_2_O/Mg^2+^ | 44.44/35.24 | 29.08/13.84 |
| root-mean-square deviation |  |  |
| Bond lengths (Å) | 0.008 | 0.005 |
| Bond angles (°) | 0.920 | 0.853 |
| Ramachandran plot % residues |  |  |
| Favored | 96.39 | 98.08 |
| Allowed | 3.61 | 1.79 |
| Outliers | 0 | 0.13 |

^a^ Values in parentheses are for the highest-resolution shell.


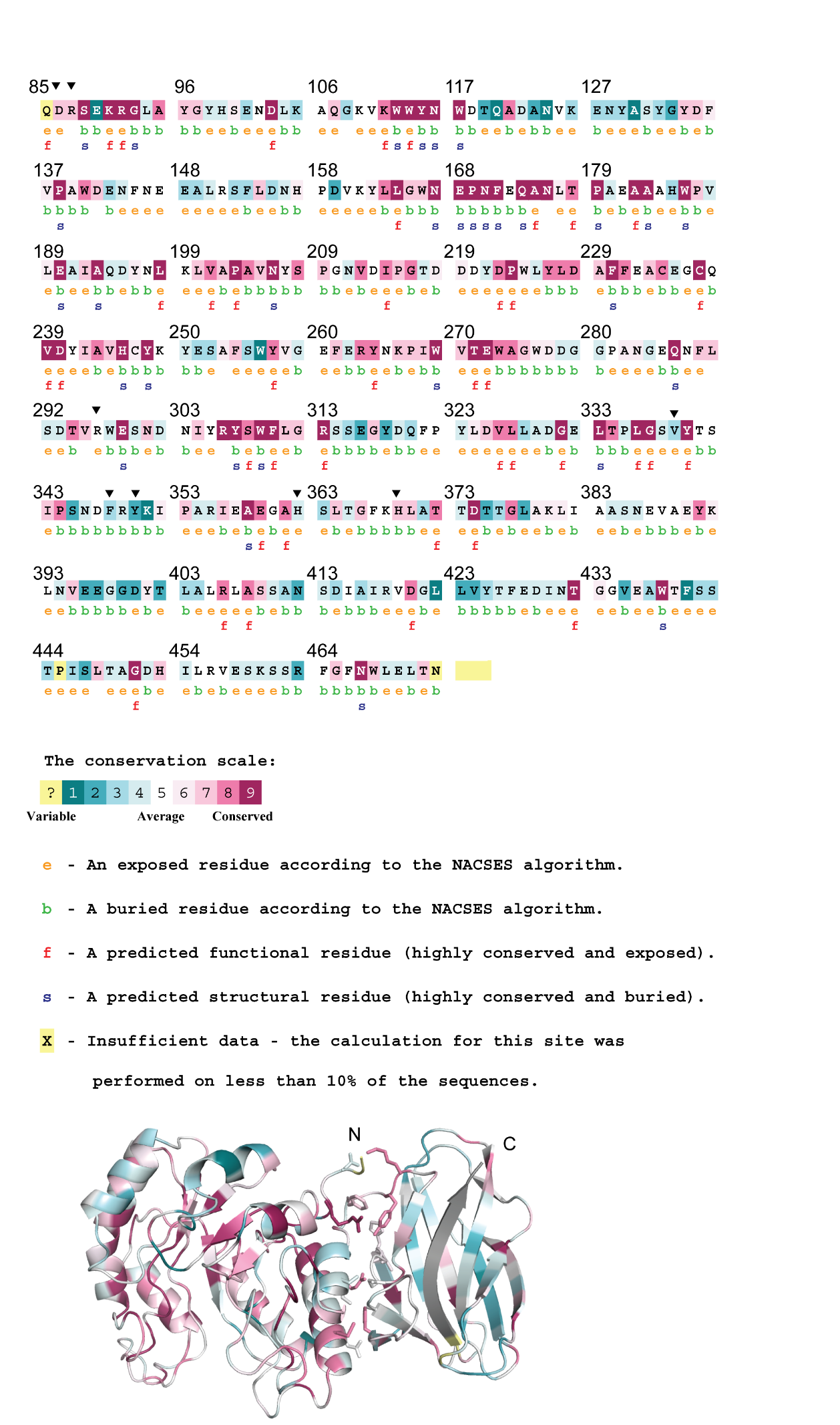


**Figure S1**. Sequence conservation analysis of the CBM6E-GH128/CBM6 protein using the ConSurf web server. The residues at the interdomain interface are labeled with black arrows in the sequence and shown as sticks in the structure.


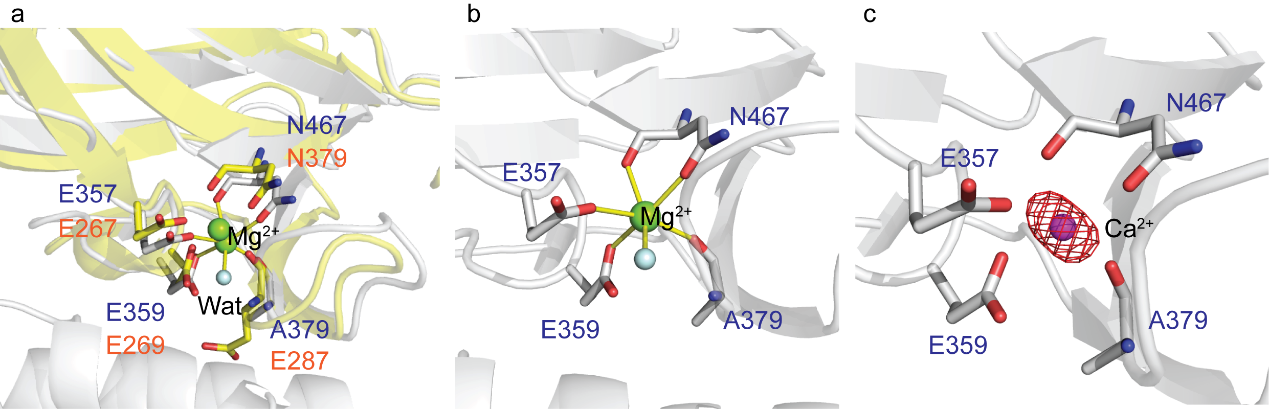


**Figure S2**. (a) Superimposition of CBM6E (gray) with ZgLamC_CBM6_ (PDB code 5fui, yellow). Residues coordinating a Mg^2+^ ion (green) within CBM6E-CBM6 and ZgLamC_CBM6_ are labeled in blue and orange, respectively. A Mg^2+^-coordinated water molecule is depicted as a cyan sphere. (b) Mg^2+^ coordination in CBM6E-CBM6 is illustrated by yellow full lines. (c) Modeling a Ca^2+^ ion into the same site resulted in a negative (red) Fo – Fc map for the ion after crystal structure refinement.


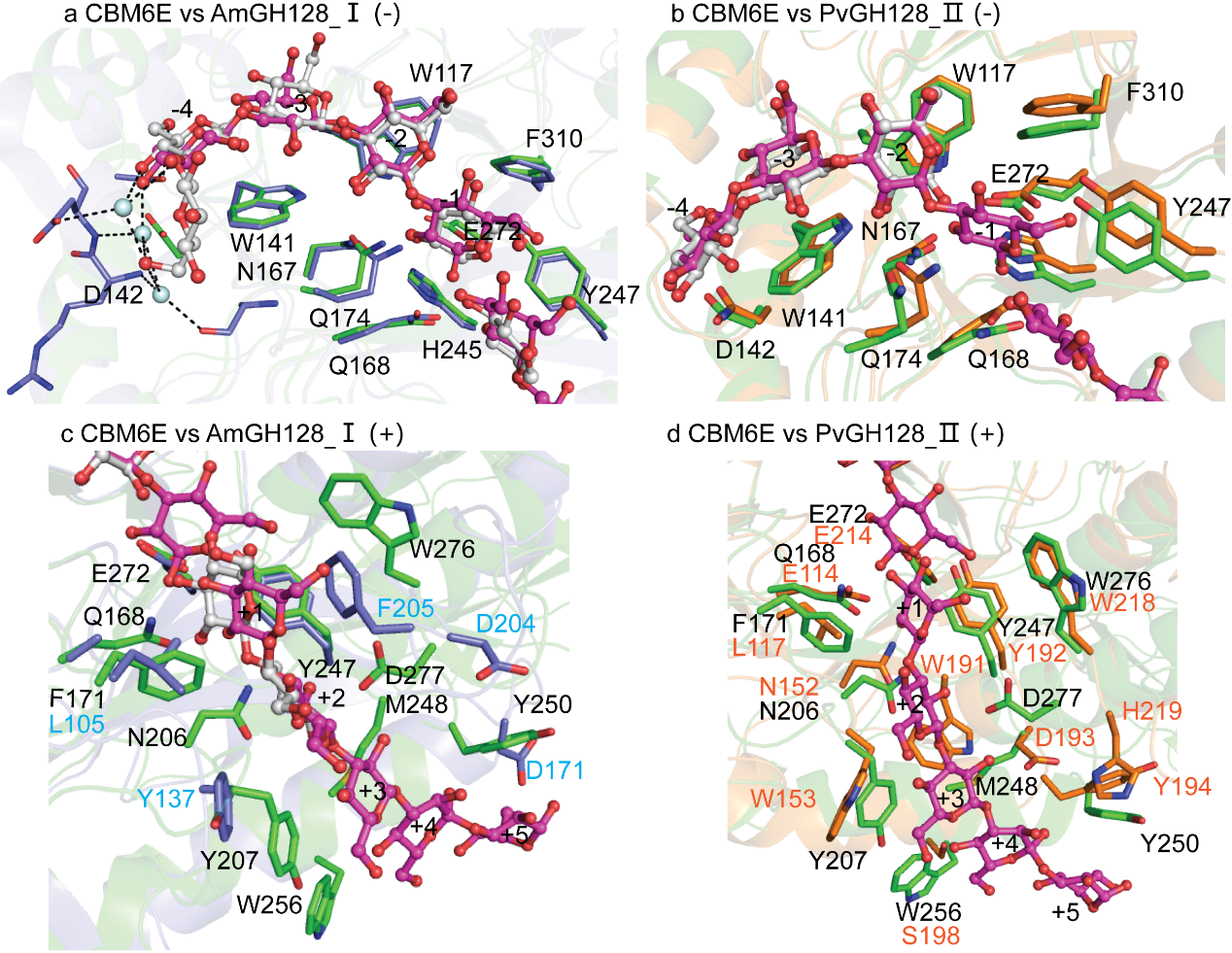


**Figure S3**. (a) Structural superposition of AmGH128_Ⅰ (PDB code 6UAS, blue) and CBM6E (green) with L5 (gray) and L4 (magenta) bound at the negative-subsite region, respectively. (b) Structural superposition of PvGH128_Ⅱ (PDB code 6UAW, orange) and CBM6E (green) with L3 (gray) and L4 (magenta) bound at the negative-subsite region, respectively. (c) Structural superposition of AmGH128_Ⅰ (PDB code 6UAU, blue) and CBM6E (green) with L2 (gray) and L5 (magenta) bound at the positive-subsite region, respectively. (d) Structural superposition of PvGH128_Ⅱ (PDB code 6UAW, orange) and CBM6E (green) with no oligosaccharide and L5 (magenta) bound at the positive-subsite region, respectively.


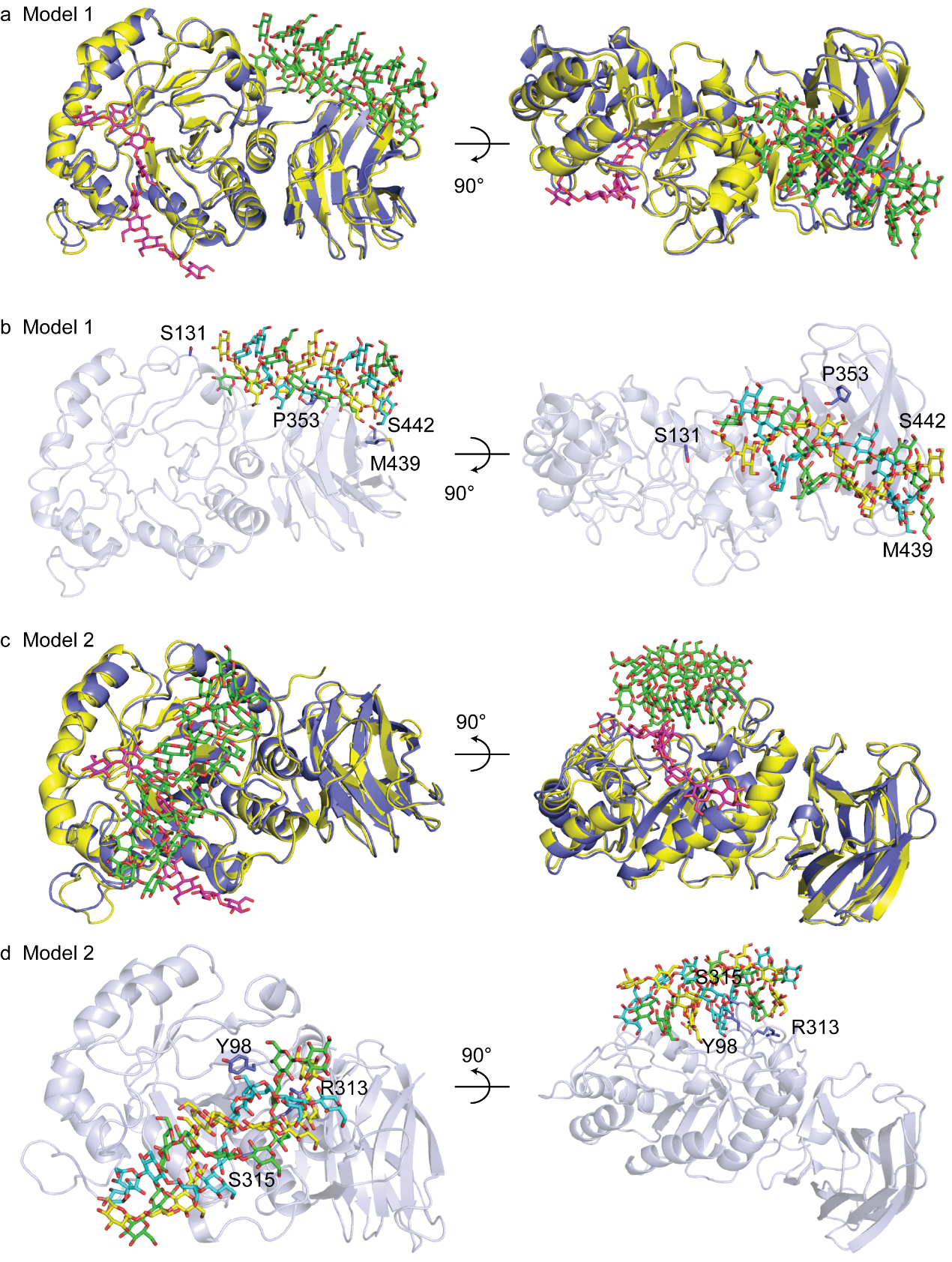


**Figure S4**. Structure superposition of CBM6E with oligosaccharides in the complex crystal structure and with a docked triple-helical β-1,3-glucan (a, c). Hydrophobic interactions between docked triple-helical β-1,3-glucans and CBM6E (b, d).


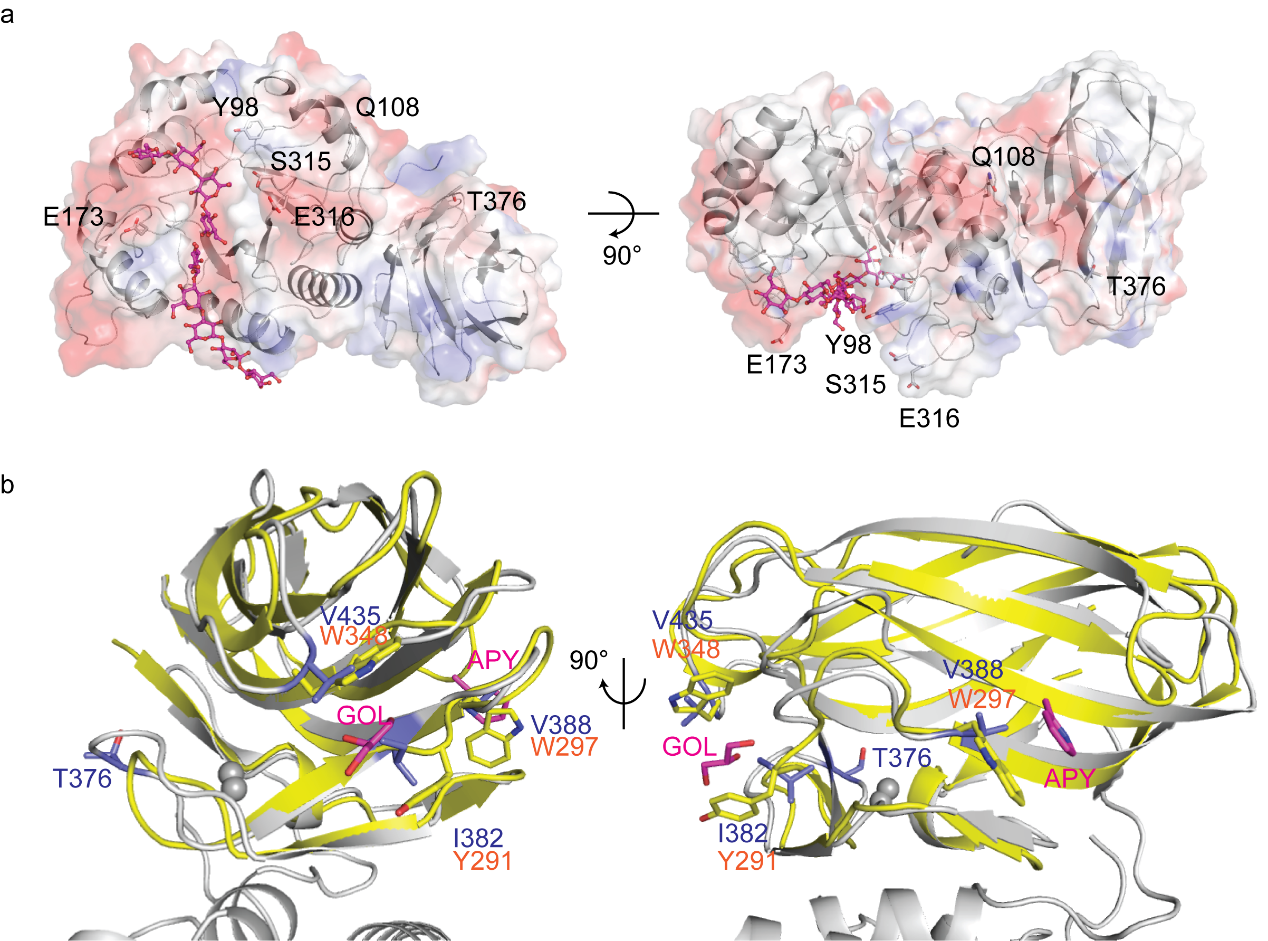
**Figure S5**. (a) Electrostatic potential surface representation of CBM6E highlighting the exposed ancillary-binding site candidates for the anchoring of curdlan according to molecular docking of triple-helical β-1,3-glucoundecamers. (b) The superimposition of CBM6E-CBM6 (gray) with ZgLamC_CBM6_ (yellow). I382 and V435 of CBM6E correspond to Y291 and W348 of ZgLamC_CBM6_, the aromatic clamp in the variable loop site (VLS) where a glycerol (GOL) is found. V388 of CBM6E corresponds to W297 of ZgLamC_CBM6_, a key sugar binding residue in the concave face site (CFS) where a 2-aminomethyl pyridine (APY) is found. T376 of CBM6E-CBM6 is not conserved in ZgLamC_CBM6_. Residues of CBM6E-CBM6 and ZgLamC_CBM6_ are labeled in blue and orange, respectively.


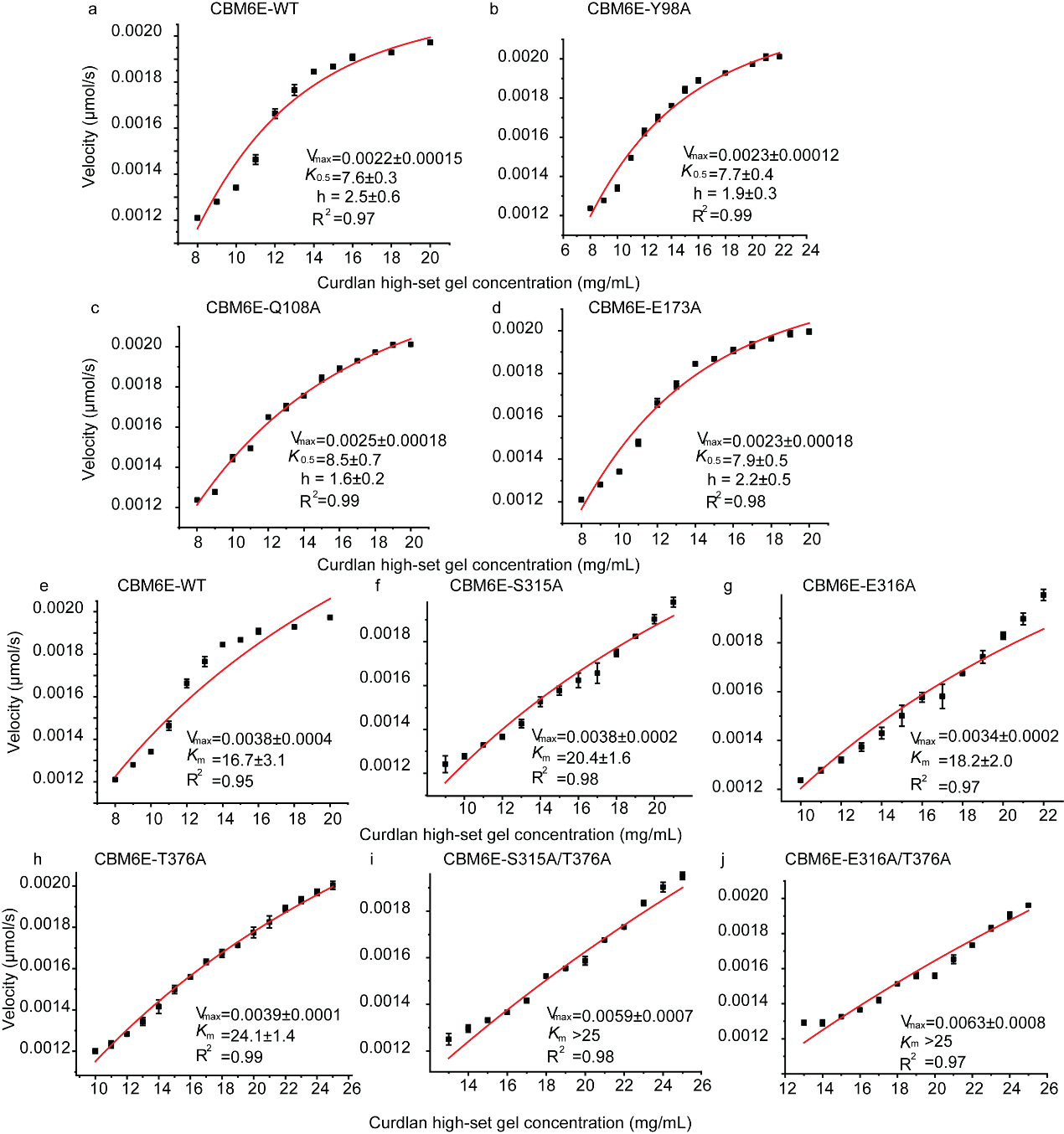


**Figure S6**. (a-d) Fitted kinetic curves of CBM6E WT, Y98A, Q108A and E173A variants using the Hill model. (e-j) Fitted kinetic curves of CBM6E WT, S315A, E316A, T376A, S315A/T376A and E316A/T376A variants using the Michaelis-Menten model.


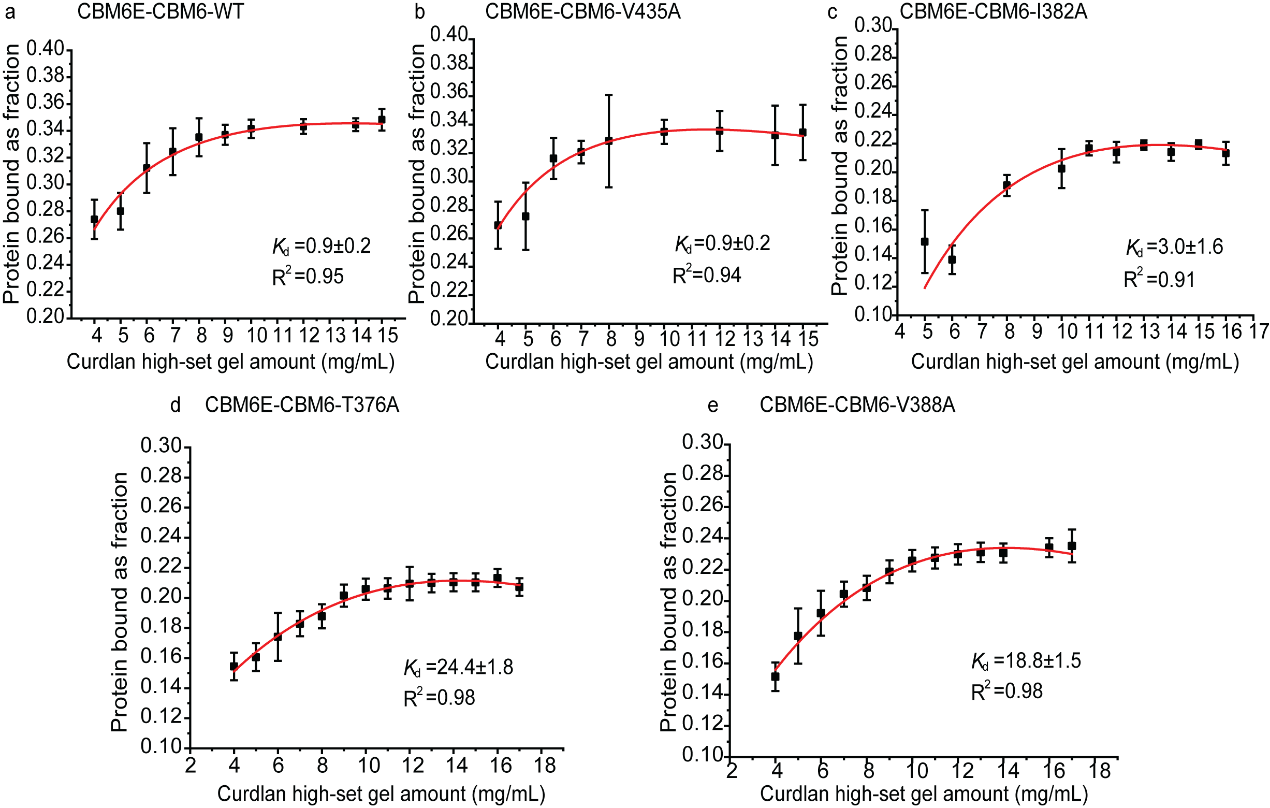


**Figure S7**. (a-e) Fractions of CBM6E-CBM6 WT and its various mutants bound versus different amounts of curdlan high-set gels were plotted and fit.


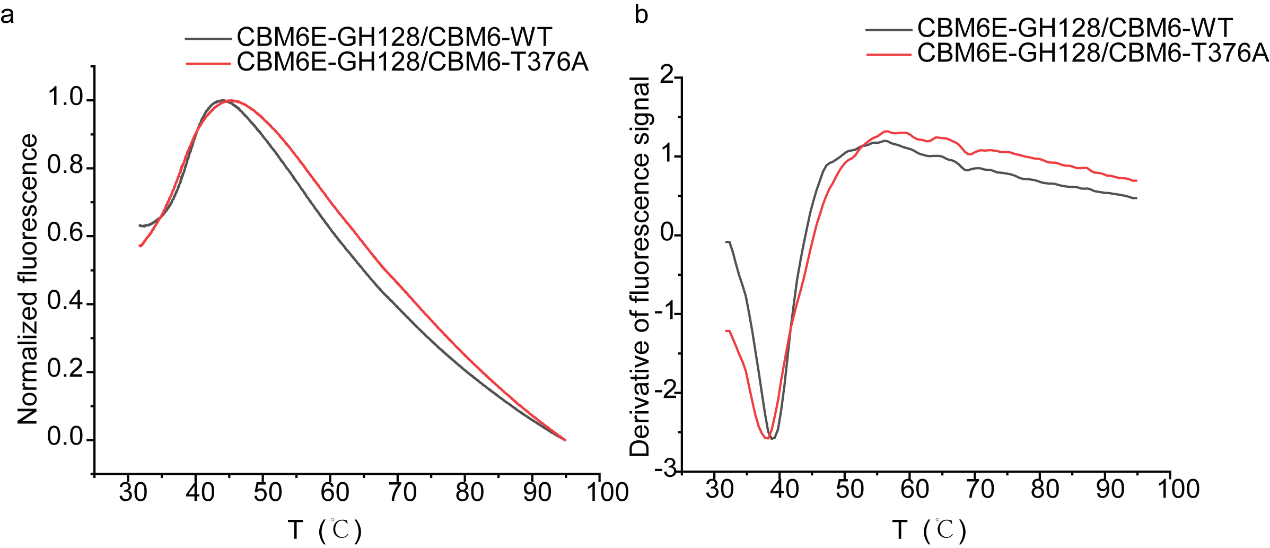


**Figure S8.** (a) Melting curves of CBM6E-GH128/CBM6 WT and its T376A mutant. (b) The first derivatives of the melting curves of CBM6E-GH128/CBM6 WT and its T376A mutant showing T_m_ values of 38.8 ℃ and 38.3 ℃, respectively.

**
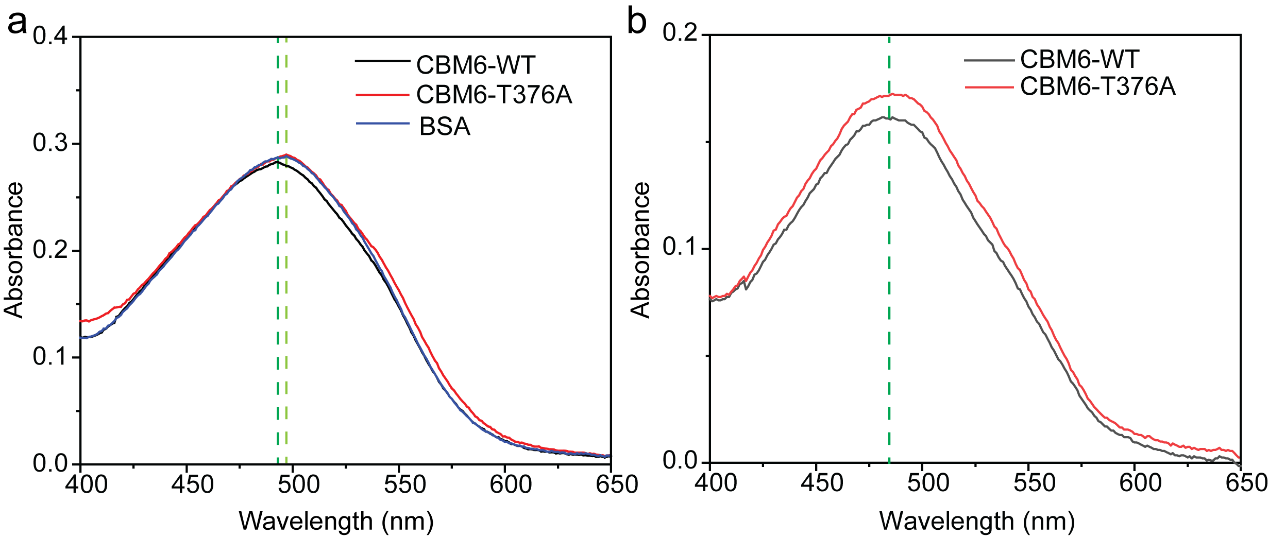
Figure S9.** Absorption spectra of the Congo red-curdlan complexes. (a) Curdlan was incubated with CBM6E-CBM6, the T376A mutant of CBM6E-CBM6 and BSA, respectively. After staining the samples with Congo red dye, the absorption spectra were recorded and plotted. (b) Absorbance of Congo red after incubation with the WT and T376A mutant of CBM6E-CBM6 at the same concentration without the addition of curdlan.
